# Nuclear instance segmentation and tracking for preimplantation mouse embryos

**DOI:** 10.1242/dev.202817

**Published:** 2024-11-06

**Authors:** Hayden Nunley, Binglun Shao, David Denberg, Prateek Grover, Jaspreet Singh, Maria Avdeeva, Bradley Joyce, Rebecca Kim-Yip, Abraham Kohrman, Abhishek Biswas, Aaron Watters, Zsombor Gal, Alison Kickuth, Madeleine Chalifoux, Stanislav Y. Shvartsman, Lisa M. Brown, Eszter Posfai

**Affiliations:** ^1^ Center for Computational Biology, Flatiron Institute – Simons Foundation, New York, NY 10010, USA; ^2^ Department of Chemical and Biological Engineering, Princeton University, Princeton, NJ 08544, USA; ^3^ The Lewis-Sigler Institute for Integrative Genomics, Princeton University, Princeton, NJ 08544, USA; ^4^ Department of Molecular Biology, Princeton University, Princeton, NJ 08544, USA; ^5^ Research Computing, Princeton University, Princeton, NJ 08544, USA

**Keywords:** Image analysis, Lineage tracking, Nuclear segmentation, Preimplantation embryo, Mouse

## Abstract

For investigations into fate specification and morphogenesis in time-lapse images of preimplantation embryos, automated 3D instance segmentation and tracking of nuclei are invaluable. Low signal-to-noise ratio, high voxel anisotropy, high nuclear density, and variable nuclear shapes can limit the performance of segmentation methods, while tracking is complicated by cell divisions, low frame rates, and sample movements. Supervised machine learning approaches can radically improve segmentation accuracy and enable easier tracking, but they often require large amounts of annotated 3D data. Here, we first report a previously unreported mouse line expressing near-infrared nuclear reporter H2B-miRFP720. We then generate a dataset (termed BlastoSPIM) of 3D images of H2B-miRFP720-expressing embryos with ground truth for nuclear instances. Using BlastoSPIM, we benchmark seven convolutional neural networks and identify Stardist-3D as the most accurate instance segmentation method. With our BlastoSPIM-trained Stardist-3D models, we construct a complete pipeline for nuclear instance segmentation and lineage tracking from the eight-cell stage to the end of preimplantation development (>100 nuclei). Finally, we demonstrate the usefulness of BlastoSPIM as pre-train data for related problems, both for a different imaging modality and for different model systems.

## Teaser

**Summary:** Image analysis pipeline for 3D nuclear instance segmentation and lineage tracking for preimplantation mouse embryos, including description of new H2B-miRFP720 reporter line and a large ground-truth dataset of nuclear instances.

## INTRODUCTION

During preimplantation development of the mouse embryo, two consecutive cell fate decisions set aside precursors of extra-embryonic tissues from cells which will form the body of the embryo. Live images of embryos expressing fluorescently tagged proteins are particularly useful for learning the rules by which cells in the embryo dynamically interact with each other to specify these fates (Chazaud and Yamanaka, 2016; Nowotschin and Hadjantonakis, 2014). However, deriving mechanistic insights from these images depends on extraction of quantitative information about cellular features, such as the position of each cell or the expression levels of specific proteins within each cell. Accurate segmentation of nuclei is a first step towards such a goal, as a cell nucleus is a good proxy for cell position relative to its neighbors and can contain information about cell-fate-specifying protein expression. To quantify these features, the segmentation must not only classify each voxel as foreground or background, but also assign each ‘instance’ (i.e. nucleus) with a distinct label (Fig. S1).

Studying the dynamics of development requires instance segmentation not for a single frame, but for a 4D series of images of a developing embryo. To observe both fate decisions in preimplantation embryos, these movies start at the early morula (eight-cell embryo) stage and end at the late blastocyst (>100-cell embryo) stage, encompassing ≈48 h of development (Chazaud and Yamanaka, 2016). Acquisition of a time lapse at sufficient spatial and temporal resolution to follow individual cells through 48 h yields nearly 200 3D images (each composed of ≈60 2D slices), containing a total of ≈8000 individual instances of nuclei (see supplementary Materials and Methods); thus, manual segmentation of every instance in every frame is not feasible. Although classical image analysis methods have had success in automated nuclear segmentation (Lou et al., 2014; Barry et al., 2022; Blin et al., 2019), these methods often require high signal-to-noise ratio (SNR) images and tuning of parameters by hand. Shallow-learning methods, like ilastik (Berg et al., 2019), offer an alternative solution for instance segmentation; however, since these methods have few trainable parameters, their performance saturates as the size of the training set grows (Berg et al., 2019). Supervised deep-learning methods have many trainable parameters; thus, the performance of these networks benefit greatly from large ground-truth sets, which allow the networks to learn salient features. Relative to other methods, deep-learning methods often generalize better across biological conditions and microscopy types (Caicedo et al., 2019).

Deep-learning methods for 3D instance segmentation of nuclei differ considerably in terms of architecture, number of trainable parameters, and post-processing steps. Because of these differences (Table S1), it is difficult to know *a priori* which method will segment nuclei most accurately for any biological system of interest. To answer this question, ground-truth data is needed to: (1) train each network on relevant image annotations and (2) to comprehensively test network performance by quantifying overlap between each instance in the ground-truth test set and each instance in the model output.

A previous study by Tokuoka et al. documented one of the first attempts to compare the performance of different deep-learning methods on a ground-truth dataset of nuclear segmentations in preimplantation mouse embryos (Tokuoka et al., 2020). Their ground-truth dataset, of nuclear centroids and semantic segmentation, spans from the two-cell stage to the 53-cell stage (Table S2) and enabled state-of-the-art performance for QCANet on early stages of development, up to approximately the 16-to-32 cell stage. Deterioration in performance of their model for later stages of development is in part due to the scarcity of training data for stages later than the 32-cell stage. The Tokuoka et al. study demonstrated a need for nuclear instance segmentation that would perform accurately up to the end of preimplantation development in live images. For example, studying the first fate decision in mammalian preimplantation development – differentiating those cells on the surface of the embryo (the trophectoderm, or TE) from those on the inside (the inner cell mass, or ICM) – requires accurate segmentation from the eight-cell stage to the ≈64-cell stage (Posfai et al., 2017). Studying the next fate decision, in which ICM cells differentiate into epiblast (EPI) and primitive endoderm (PE) cell populations that spatially segregate, requires accurate segmentation for later stages (>100 nuclei) (Saiz et al., 2016; Yamanaka et al., 2010; Chazaud and Yamanaka, 2016).

To this end, here we first generate a mouse line that expresses a near-infrared nuclear reporter H2B-miRFP720. H2B-miRFP720 is well suited for live imaging owing to its reduced phototoxicity and its lack of spectral overlap with reporters in the visible range. We generate a large dataset, called BlastoSPIM (1.0), of light-sheet images of H2B-miRFP720-expressing preimplantation embryos with corresponding ground truth for nuclear instance segmentation. Our BlastoSPIM dataset provides a foundation for evaluating both new and existing methods for nuclear instance segmentation, just as other large ground-truth datasets have historically enabled scientific progress by allowing researchers to focus on the methods rather than the data collection and annotation. We use this dataset – that extends from the two-cell stage to the >100-cell stage – to train and test seven different deep-learning methods: Cellpose, Stardist-3D, RDCNet, U3D-BCD, UNETR-BCD, QCANet, and ELEPHANT. The Stardist-3D model, trained on BlastoSPIM 1.0, achieves state-of-the-art performance, detecting nuclei with high accuracy in early- to mid-stage preimplantation embryos. Next, to improve segmentation accuracy at later embryonic stages, we generate a new ground-truth dataset, termed BlastoSPIM 2.0, on blastocyst embryos and show that Stardist-3D trained on this dataset achieves similarly high nuclear segmentation accuracy, even for embryos with >100 cells. By using the two (early and late) Stardist-3D models, we develop a complete pipeline for automatic segmentation, segmentation correction, nuclear centroid registration, lineage tracking, and extraction of nuclear-localized signals. By enabling the tracking of nuclei from the eight-cell stage to the >100-cell stage, this pipeline provides insight into the dynamics of nuclear volumes and nuclear shapes with respect to cell cycle and cell fate (ICM-TE). Analysis of time-lapse images of a mouse line expressing both H2B-miRFP720 and Cdx2-eGFP (McDole and Zheng, 2012) reveals the dynamic coupling between cell cycle progression and Cdx2 expression, not only during the 16-cell stage (McDole et al., 2011), but also for the subsequent cell cycle. We close by demonstrating that our ground-truth dataset and corresponding models aid nuclear segmentation in other model systems [intestinal organoids (De Medeiros et al., 2022) and *Platynereis dumerilli* embryos (Lalit et al., 2022)] as well as in data from other imaging modalities (spinning disk confocal).

## RESULTS

### Establishment of a near-infrared nuclear reporter mouse line

**Fig. 1.**
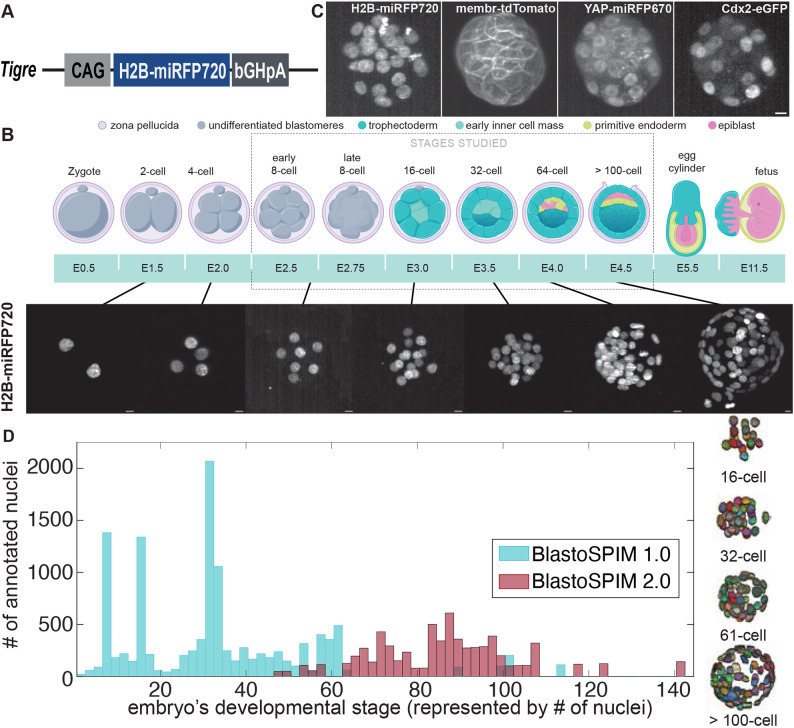
**BlastoSPIM datasets, ground truth of nuclear instance segmentation for embryos expressing a new near-infrared nuclear marker.** (A) Schematic of targeted TIGRE locus with the CAG-H2B-miRFP720 insert. (B) Top: cartoon of preimplantation mouse development. After fertilization, the zygote undergoes cleavage divisions. At the eight-cell stage, compaction and polarization occur. By the 32-cell stage, a subset of cells called the trophectoderm (TE) form the surface of the embryo; the remaining cells form the inner cell mass (ICM). By the 64-cell stage, the ICM cells begin to pattern into two fates, primitive endoderm (PE) and epiblast (EPI), which are spatially segregated by implantation at the >100-cell stage. Bottom: maximum-intensity projected images, acquired with SPIM, of preimplantation embryos expressing H2B-miRFP720 at different developmental stages. (C) Preimplantation embryo expressing four spectrally distinct fluorescent reporters: H2B-miRFP720, mTmG, YAP-emiRFP670, Cdx2-eGFP. Maximum intensity projections of images acquired with SPIM. (D) Number of nuclei per embryonic stage (represented by embryo cell number) for both BlastoSPIM 1.0 (blue, for initial benchmarking of methods) and BlastoSPIM 2.0 (red, for extending accurate segmentation to later stages). Right: ground truth of nuclear segmentation for four embryos from different stages. Scale bars: 10 µm.

Multicolor imaging is key to simultaneous recording of morphogenesis and cell fate specification. To enable visualization of cell nuclei in concert with various other molecular markers, which are typically tagged with green, red or far-red fluorescent proteins, we generated a new spectrally distinct near-infrared nuclear mouse line expressing H2B-miRFP720 (Fig. 1A,B). First, using 2C-HR-CRISPR (Gu et al., 2018) we targeted CAG H2B-miRFP720 to the TIGRE locus (Concordet and Haeussler, 2018). Early preimplantation embryos from this line showed uniform H2B-miRFP720 expression; however, by the mid blastocyst stage significant dimming of the fluorescent signal was noted, even in freshly isolated embryos. A second mouse line harboring CAG ORF-2A-H2B-miRFP720 in the TIGRE locus did not, however, exhibit the same dimming issue, and rather showed a slight increase in H2B-miRFP720 intensity during preimplantation development. We therefore used two sgRNAs to delete the ORF-2A in this line, resulting in a CAG H2B-miRFP720 line (hereafter referred to as the H2B-miRFP720 mouse line) with bright reporter expression across all preimplantation stages (Fig. 1A,B). miRFP720 can be readily multiplexed with far-red fluorescent reporters such as emiRFP670 or the Halo-tag visualized with the JF646 dye – therefore this mouse line allows simultaneous imaging of up to four different reporters in mouse embryos (Fig. 1C). Furthermore, the long wavelength used for its detection makes H2B-miRFP720 ideal for deep-tissue imaging and results in reduced phototoxicity. We verified that neither the presence of the H2B-miRFP720 transgene, nor ≈48 h live imaging, affects preimplantation development and lineage segregation (Fig. S2). Specifically, we compared incubator-cultured [from embryonic day (E) 2.5 to E4.5] wild-type (WT) and H2B-miRFP720 embryos, as well as unimaged and imaged H2B-miRFP720 embryos, by immunostaining for lineage markers Nanog (EPI) and Sox17 (PE) following culture or imaging. We found no statistically significant change in total cell number and comparable development of all three lineages (EPI, PE and TE) for all comparisons examined.

### A novel ground-truth dataset of preimplantation mouse embryos for comparing nuclear segmentation methods

Using selective plane illumination microscopy (SPIM) we acquired 3D live images of H2B-miRFP720-expressing preimplantation embryos at various developmental stages. We created a new ground-truth dataset with full 3D nuclear instance segmentation. This dataset, which we call BlastoSPIM 1.0 (concatenation of blastocyst and SPIM), is one of the largest and most complete of its kind (Table S2) with more than 570 high-resolution images and ≈12,000 nuclei annotated, spanning all preimplantation stages (Fig. 1D) (see Materials and Methods, Dataset characteristics; Fig. S3). The quality, detail, and size of the BlastoSPIM dataset makes it unique relative to other publicly available ground-truth datasets for nuclear instance segmentation (Table S2).

To quantitatively illustrate the challenges posed by densely packed nuclei for instance segmentation, using BlastoSPIM 1.0, we calculated how nucleus-to-nucleus distances change from the 16-cell stage to the >100-cell stage. The surface-to-surface distance between nearest-neighbor nuclei has a median of 6.0 µm at the 16-cell stage, 2.9 µm at the 32-cell stage, 1.8 µm at the 64-cell stage, and ≈0.5 µm at the >100-cell stage (Fig. S4). This decrease in nearest-neighbor distance is not accompanied by a comparable decrease in nuclear size (Fig. S4); thus, instance segmentation is expected to be considerably more challenging as development progresses.

In addition, the characteristics of image acquisition often complicate segmentation. For example, live images often have low foreground intensities relative to background because the exposure of embryos to light has to be limited to prevent phototoxicity (Laissue et al., 2017). Moreover, the sample is imaged along a single axis (*z*-axis, by convention), resulting in voxel anisotropy, poorer *z*-resolution than *xy*-resolution. Our ground-truth dataset contains a range of foreground-background intensity differences (see Materials and Methods, Dataset characteristics; Fig. S3) and has a voxel anisotropy of ≈10. In summary, because of its size as well as its diversity in developmental stage and foreground-background intensity difference, our dataset of manually annotated 3D instances of nuclei is uniquely suited to interrogate the performance of instance segmentation methods for nuclei in preimplantation embryos.

### Benchmarking of seven instance segmentation methods on BlastoSPIM 1.0 reveals superior performance of Stardist-3D

We used our ground-truth dataset to compare seven instance segmentation networks (Table S1): Cellpose (Stringer et al., 2021), Stardist-3D (Weigert et al., 2020), RDCNet (Ortiz et al., 2020), U3D-BCD (Lin et al., 2021), UNETR-BCD (Hatamizadeh et al., 2022), ELEPHANT (Sugawara et al., 2022), and QCANet (Tokuoka et al., 2020). These methods span a variety of network architectures, from those including recurrent blocks or transformers to more conventional U-Nets. They also represent the instances in different ways. For example, Stardist-3D computes a set of distances to the boundary, whereas Cellpose predicts gradients that are tracked to the instance center.

**Fig. 2.**
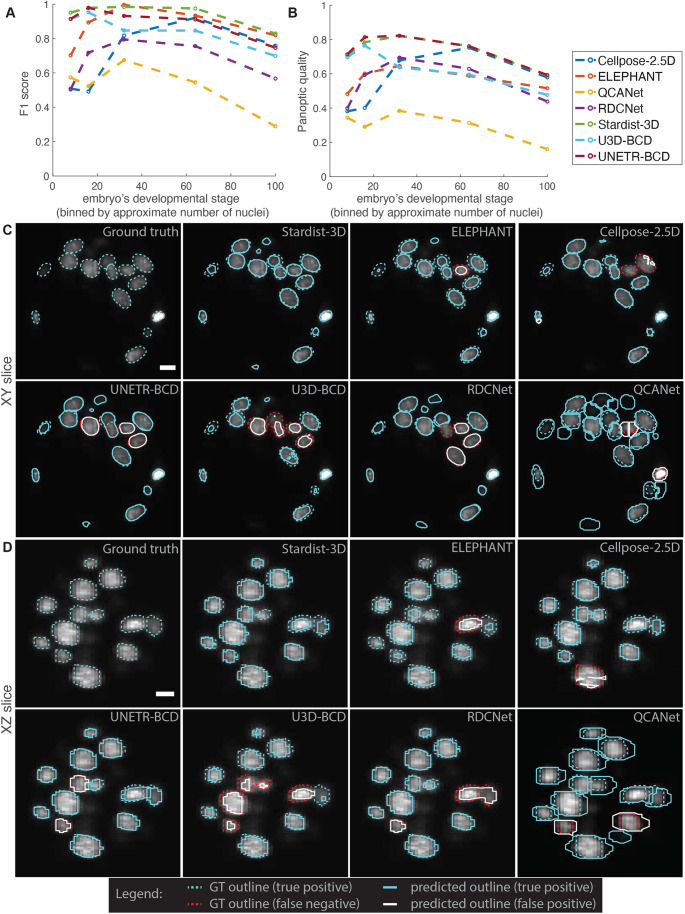
**Evaluation of seven instance-segmentation networks trained on BlastoSPIM 1.0 across preimplantation developmental stages.** (A,B) F_1_-score (A) and panoptic quality (B) across developmental stage. See Figs S5 and S6 for evaluation at different intersection-over-union (IoU) cutoffs. (C,D) Qualitative evaluation on a 60-cell embryo. Instance contours overlaid on a representative slice of the intensity image in *xy* (C) and in *xz* (D). Each panel is labeled as ground-truth or the model evaluated. Outlines of ground truth, true positives (blue), false positives (white) and false negatives (red) are shown for each model. Ground truth indicated by dashed line, model predictions indicated by solid line. False positives and false negatives are defined by comparing the 3D instance segmentation results rather than the results shown in a single 2D slice. Note that if the model-predicted instance does not overlap sufficiently well with the ground-truth instance, the result is a false positive and a false negative. In C, the extra cyan outlines for QCANet are predicted instances which match with instances in nearby *z*-slices but over-extend in *z*. See Tables S3 and S4 for evaluation of model failures. Scale bars: 10 µm.

We trained each model with data from 482 3D images of embryos from BlastoSPIM 1.0 and then evaluated on a test set composed of moderate foreground-background intensity difference data. To interrogate stage-specific performance, we divided this test set into developmental stages such that it contained ≈120 nuclei from each stage (e.g. more images from earlier stages than later stages). To benchmark each method, we compared the ground-truth instances and model-inferred instances by computing matches based on the intersection-over-union (IoU). A model-inferred instance is considered as a match to a ground-truth instance if the IoU is at least 0.5 (see different cutoffs in Figs S5 and S6). Based on this matching, we computed the *F*_1_ score, defined as 
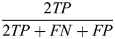
, where TP, FP, and FN are the number of true positives, false positives, and false negatives, respectively (Fig. 2A). We also quantified the accuracy of the model predictions based on the panoptic quality (see Materials and Methods, Dataset splits and evaluation metric; Fig. 2B). As opposed to the *F*_1_ score, which counts matches in a binary way based on a threshold in IoU, the panoptic quality also depends on the sum of the IoUs that are above the specified IoU threshold.

Based on the *F*_1_ score, the Stardist-3D model outperformed all other methods across developmental stages (Fig. 2A). From the eight-cell stage up to the 64-cell stage, the *F*_1_ score remained above 95%. This is significantly higher than the state-of-the-art results on similar (confocal) data from preimplantation mouse embryos, particularly in embryos with >32 nuclei (Tokuoka et al., 2020). By comparison to the Stardist-3D strong *F*_1_ score across stages, the *F*_1_ score of the other methods depended more strongly on developmental stage (Fig. 2A). For example, both UNETR-BCD and the related U3D-BCD method performed reasonably well at the eight- and 16-cell stages but were unable to detect several nuclei in later stages as nuclei became more densely packed. By contrast, the performance of Cellpose and RDCNet slightly improved from the eight-cell stage to the 64-cell stage, then decreased at the later stages.

Stardist-3D also achieved the highest panoptic quality, approximately equal to that of UNETR-BCD, across developmental stages (Fig. 2B). Despite the UNETR-BCD low *F*_1_ as compared to both Stardist-3D and ELEPHANT, its high panoptic quality can be explained as follows: the IoUs for UNETR-BCD successful matches to the ground-truth instances are high even though it has fewer matches. By contrast, although ELEPHANT had a reasonably high *F*_1_ score for the 16-cell and later stages, the panoptic quality remained relatively low across stages. This is likely explained by the ELEPHANT constraint that nuclei be represented only as ellipsoids; by contrast, Stardist-3D's representation of nuclei as star-convex polyhedra approximates well the nuclear shapes in our ground-truth dataset (Fig. S7).

At very stringent IoU cutoffs, the *F*_1_ score and panoptic quality for Stardist-3D drop below that of UNETR-BCD (see Fig. S6). These low *F*_1_ score and panoptic quality likely reflect the limitations of parameterizing ground-truth nuclear shapes as star-convex polyhedra with a fixed number of rays (Fig. S7). Despite this performance of UNETR-BCD at high IoU cutoffs, we conclude that the performance of Stardist-3D strikes an important balance: achieving the highest *F*_1_ score at IoU cutoffs that are high enough (≈0.5) for the purposes of nuclear tracking and subsequent analysis (see Results, BlastoSPIM-trained Stardist-3D models enable lineage tracking).

In terms of both performance metrics, one method, QCANet, performed worse than the other methods across various IoU cutoffs. Two key factors likely contributed to this poor performance. First, QCANet makes the images isotropic by decreasing the *xy*-resolution and interpolating in *z*, and this coarser resolution likely complicates the prediction of the boundary between closely juxtaposed nuclei. Second, since QCANet uses centroid-based watershed, errors in the predicted centroid locations impact the predicted boundaries between instances and can give rise to instances with unrealistic shapes.

Fig. 2C,D shows qualitative results of these seven networks on an embryo with 60 nuclei based on two 2D image slices (one in *xy*, the other in *xz*). On this test image, Stardist-3D achieved the best F1-score by producing only one false negative (not shown in slice) and no false positives. Although ELEPHANT produced one instance per ground-truth instance, two of those instances overlapped poorly with the corresponding ground truth; these resulted in two false negatives and two false positives. UNETR-BCD and Cellpose each produced approximately ten errors, including false positives and false negatives. The remaining three methods produced significantly more errors than the others on this test case. Below we summarize the typical errors made by each network.

U3D-BCD, UNETR-BCD, and RDCNet missed several nuclei due to undersegmentation, the merging of more than one nucleus into the same instance (see Fig. 2C for UNETR-BCD and Fig. 2D for RDCNet). On the other hand, Cellpose often oversegmented nuclei into small spurious instances or missed a ground truth instance entirely (see Fig. 2C). QCANet often predicted the right number of nuclear centroids, and thus avoided over- or undersegmentation, but if two predicted centroids fell within the same contiguous region of the semantic segmentation, the watershed-based post-processing often split the mask improperly (see neighboring false positives in Fig. 2C).

Some methods produced instances with shapes that are inconsistent with the range of ground-truth nuclear shapes. For example, QCANet overpredicted nuclear sizes (see instances which extend across more *z*-slices than the ground-truth instances in Fig. 2D). By contrast, Cellpose and RDCNet tended to produce some small false positives with irregular shapes (Fig. 2C,D). U3D-BCD tended to produce instances with holes or gaps (Fig. 2C). Although models that make few hard assumptions about nuclear shape sometimes performed best on particularly difficult cases like mitotic nuclei or polar bodies (Tables S3 and S4), our results suggest that the methods which make biologically plausible assumptions about nuclear shape (ELEPHANT and Stardist-3D) often achieve higher *F*_1_ scores. In summary, our comprehensive benchmarking of different models provided insight into the strengths and weaknesses of each network and identified Stardist-3D as the best performing network for 3D images of live preimplantation embryos. Moreover, our large ground-truth dataset can be used to test whether future neural network architectures can outperform Stardist-3D in nuclear instance segmentation for preimplantation mouse embryos.

### Extending the Stardist-3D segmentation accuracy up to the >100-cell stage

Although Stardist-3D performed well up to the 64-cell stage (Fig. 2C,D), its performance deteriorated at later stages. Therefore, we set out to improve accuracy by training the network on late-stage ground-truth data. We hand-annotated an additional 80 3D images of late-stage embryos expressing H2B-miRFP720, containing more than 6600 nuclear instances – a dataset we termed BlastoSPIM 2.0 (Fig. 1D). We trained and validated a new Stardist-3D model based on 72 images of late blastocysts from BlastoSPIM 2.0 (termed ‘late blastocyst model’).

**Fig. 3.**
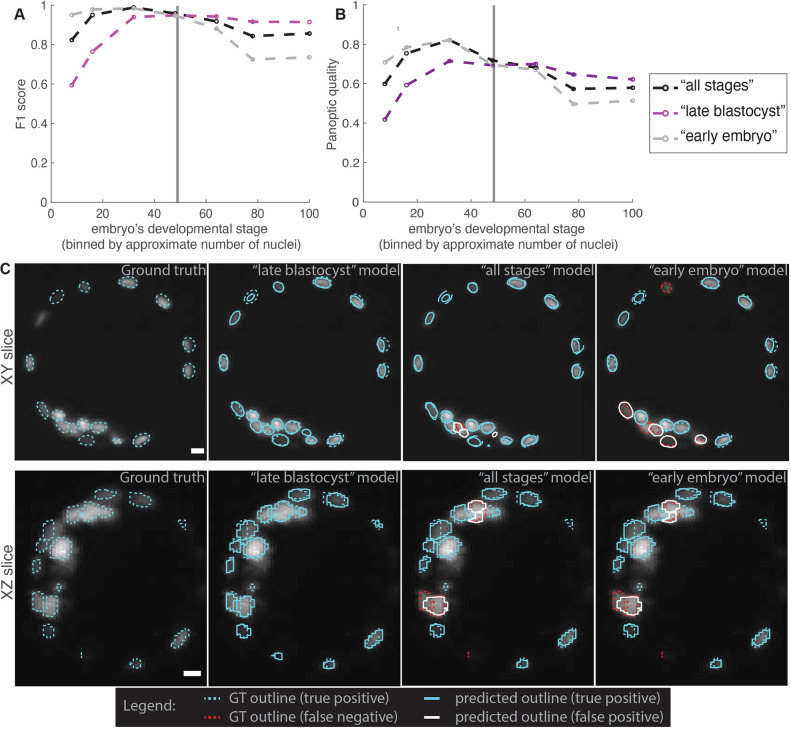
**Comparing models trained on BlastoSPIM 1.0 (early embryo model), BlastoSPIM 2.0 (late blastocyst model), and on both 1.0 and 2.0 (all stages model).** (A,B) F_1_-score (A) and panoptic quality (B) for the moderate foreground-background intensity difference test set. See Figs S8 and S9 for evaluation at different intersection-over-union (IoU) cutoffs. Vertical line: approximate developmental stage at which to transition from the early embryo model to the late blastocyst model. (C) Qualitative evaluation on a 106-cell embryo. Instance contours overlaid on a representative slice of the intensity image in *xy* (top) and in *xz* (bottom). Each panel is labeled as either ground truth or the model evaluated. Outlines of ground truth, true positives (blue), false positives (white) and false negatives (red) are shown for each model. Ground truth indicated by dashed line, model predictions indicated by solid line. Note that if the model predicted instance does not overlap sufficiently well with the ground-truth instance, the result is a false positive and a false negative. See Fig. S10 for errors produced by inference of the late blastocyst model on early embryo data. Scale bars: 10 µm.

The late blastocyst model outperformed the previous Stardist-3D model (hereafter referred to as the ‘early embryo model’) from Fig. 2 on test images of late blastocysts, and underperformed it on test images of embryos with fewer than 64 nuclei (Fig. 3A,B; Figs S8, S9, S10). In particular, the late blastocyst model performed better than the early embryo model in cases with closely juxtaposed nuclei (Fig. 3C; Fig. S11), while it often oversegmented polar bodies, which typically only appear in images of early embryos (Fig. S10). For comparison to these two models, we also trained a model on both BlastoSPIM 1.0 and 2.0 (‘all stages model’) and found that the performance of this model fell between the performance of the other two Stardist-3D models (Fig. 3A,B; Figs S8 and S9). As a result, at any developmental stage, one would achieve higher performance with the stage-specific models (see performance at higher IOU cutoffs in Fig. S9). Based on our analysis in Fig. 3A,B, we transitioned from using the early embryo model to using the late blastocyst model when the embryo has ≈48 nuclei (in the 32-to-64 cell transition).

To address whether the early embryo model and late blastocyst model would perform well even for cases of low foreground-background intensity difference, we evaluated these Stardist-3D models on such a test set (Fig. S11), separate from the test set in Fig. 2. Although only a third of the images in the training set for the early embryo model met our definition of low foreground-background intensity difference (Fig. S3), the *F*_1_ score of the early embryo model for the low foreground-background intensity difference test set was ⪆90% up to the 64-cell stage. For later stages, the late blastocyst model outperformed the early embryo model by achieving an *F*_1_ score of ≈86%. Since the images in our low foreground-background intensity difference test represent the most challenging cases, where even annotation by human experts is difficult (Fig. S11D), we expect our models to generalize to long-term live-imaging datasets with moderate foreground-background intensity difference.

### BlastoSPIM-trained Stardist-3D models enable lineage tracking

**Fig. 4.**
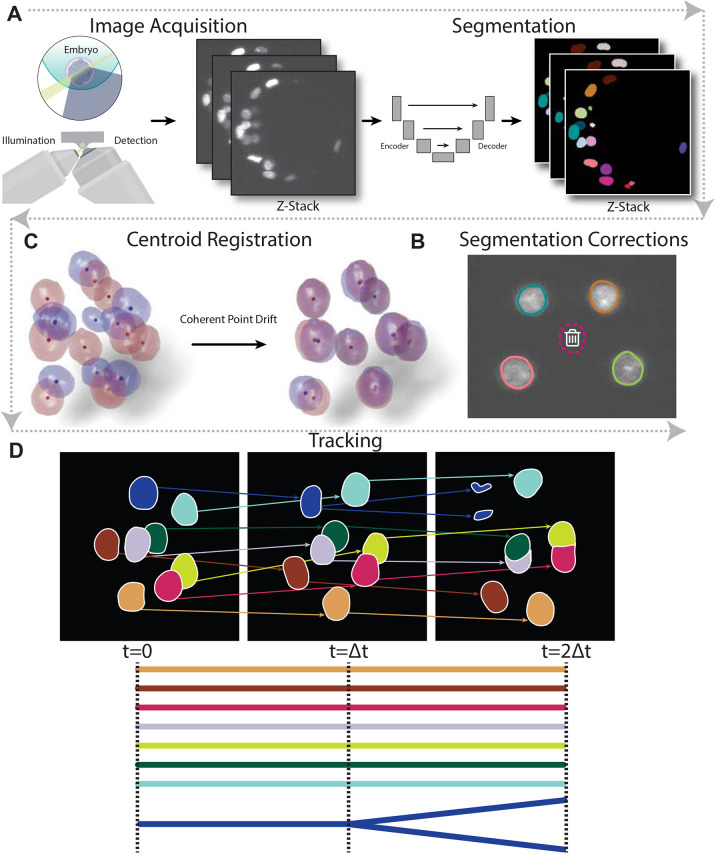
**Analysis pipeline, from image acquisition to lineage tree construction.** (A) Image acquisition and segmentation. Time series of 3D light-sheet images were segmented automatically using the early embryo and the late blastocyst Stardist-3D models. Green, light sheet for illumination; blue, emitted light collected by the detection objective. (B) Segmentation corrections. Errors in the segmentation were hand-corrected by overlaying the segmentation with the raw image in each frame and removing false positives and/or adding instances which were missing. (C) Centroid registration. Using the instance centroids extracted from the corrected segmentation, consecutive pairs of frames are aligned to account for rigid motion the embryo experiences during image acquisition. (D) Lineage tracking. After registration, a set of lineage trees was constructed (one for each of the nuclei in the first frame) by matching nuclear identities between pairs of consecutive frames. Gray arrows indicate order of steps in the pipeline.

By integrating the nuclear segmentation results from the BlastoSPIM-trained Stardist-3D models with lineage tracking, we developed a complete image analysis pipeline for live images of preimplantation development. The basic steps of the pipeline outlined below have been implemented in both a command line and jupyter notebook interface, with tutorials and an example dataset (see Data availability). First, we acquired a time series of 3D images of an H2B-miRFP720-expressing embryo, with one *z*-stack acquired every 15 min. We segmented the images by either the early embryo or late blastocyst model, switching models at around the 48-cell stage (Fig. 4A). As accurate lineage tracking is contingent upon accurate segmentation, we hand-corrected remaining errors using AnnotatorJ (Hollandi et al., 2020). AnnotatorJ overlays each segmented region of interest (ROI) onto the original image and provides an intuitive interface to edit, delete, or create ROIs. Since AnnotatorJ was originally designed for 2D images, we introduced several enhancements for corrections of 3D segmentations. Segmentation errors were corrected by removing false positives, adding missing instances, and editing oversegmented regions.

**Fig. 5.**
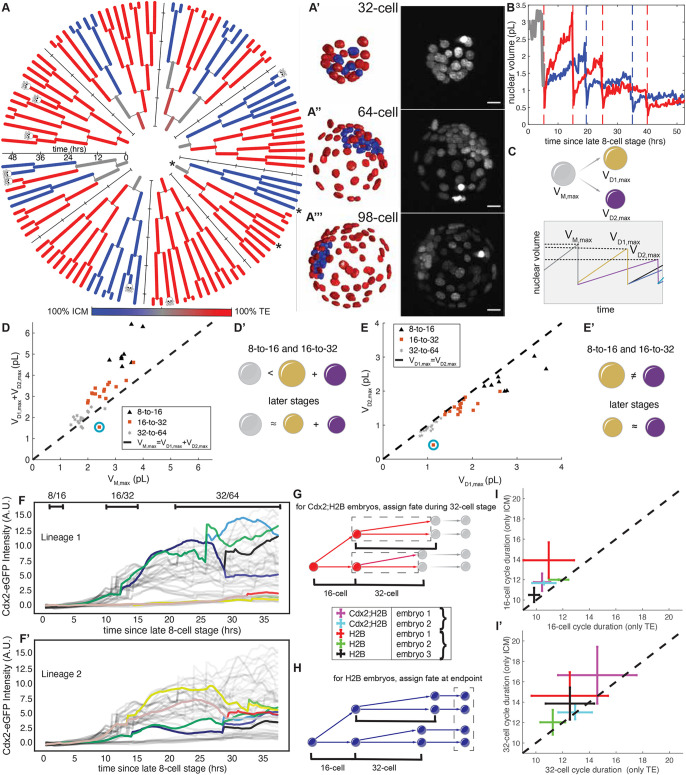
**Nuclear shape dynamics, cell cycles, and fate specification.** (A) Lineage trees. Color indicates eventual contributions to the inner cell mass (ICM) and trophectoderm (TE). Skull indicates a cell death event. Paths between the asterisked root and two asterisked leaves shown in B. (A′-A‴) Maximum intensity projections of H2B signal and segmentations, colored as in A. (B) Nuclear volumes along the paths indicated by asterisks in A. Dashed lines indicate division events. (C) Cartoon of nuclear volume dynamics along the lineage tree. Gray, gold, and purple show mother nucleus, larger daughter nucleus, and smaller daughter nucleus, respectively. (D,D′) Comparison of summed daughter nuclear volumes to that of the mother. (E,E′) Comparison of volumes of two daughter nuclei. In D and E, the cyan circle shows an outlier due to nuclear fragmentation. (F,F′) Cdx2-eGFP dynamics in lineages from two different eight-cell-stage nuclei (see also Fig. S18). Gray lines show Cdx2-eGFP traces from other nuclei in the same embryo. The colored lines represent the Cdx2-eGFP traces for all nuclei descending from a common nucleus at the eight-cell stage. Whenever a division occurs within the lineage, a new colored line emerges. For each branch, transcription factor intensities within every cell cycle were smoothed using a rolling average over a 2.5 h window. (G) Schematic of a Cdx2^+^ lineage, assigned based on Cdx2-eGFP levels during 32-cell stage. (H) Schematic of an ICM lineage, assigned based on nuclear position at endpoint. (I,I′) Comparison of mean (±s.d.) of the 16-cell (I) and 32-cell (I) cycle durations for purely ICM-contributing nuclei and purely TE-contributing nuclei. For boxplots, see Figs S16 and S17. Scale bars: 20 µm.

To track nuclear instances across time, we corrected for movement of the embryo during image acquisition by computing a rigid transformation which aligns consecutive pairs of frames. Based only on nuclear centroids, we registered consecutive frames by using the coherent point drift algorithm from Myronenko and Song (2010). Finally, we performed semi-automated lineage tracking on the registered nuclear instances. Our algorithm operates sequentially, matching nuclei to their predecessors in the previous frame. Non-dividing nuclei were tracked using nearest-neighbor association between instance centroids. Additionally, for dividing nuclei, we used a heuristic based on the difference in nuclear volume between potential mother-daughter triples. To quantify the performance of our tracking method, we compared the tracks generated by our method to ground-truth corrected tracks for the time-lapse image in Fig. 5A-A‴. Based on false negative and false positive edges in the lineage trees, we calculated an *F*_1_ score of 0.996. By contrast, a widely used method called Trackmate (Tinevez et al., 2017) achieved an *F*_1_ score of 0.971 when it was provided with preregistered segmented images and an *F*_1_ score of 0.646 with unregistered segmented images.

After lineage construction, an additional module in the pipeline optionally extracts the intensities of nuclear-localized factors over the full lineages. If the additional imaging channel(s) includes fluorescently tagged transcription factors (TFs), this module enables the analysis of the dynamics of fate specification over the lineage trees.

### Characterizing the dynamics of nuclear volumes, cell cycles, and fate specification from the eight-cell to late blastocyst stage

We used the pipeline (Fig. 4) for the semi-automated analysis of an embryo from the eight-cell stage to the ≈100-cell stage (Fig. 5A-A‴; Fig. S12). After ≈52 h of development, the embryo reached the 98-cell stage. At the final time point, we used nuclear position as a proxy to assign ICM and TE fates and found 27 ICM and 71 TE nuclei, in keeping with fate proportions previously reported (Saiz et al., 2016). To our knowledge, these are some of the furthest tracked cell lineages in preimplantation development.

Next, we characterized changes in nuclear volumes and shapes in individual cell lineages in Fig. 5A (see Materials and Methods, Quantifying nuclear volume and aspect ratio). First, we examined how accurately our BlastoSPIM-trained models report on these features by comparing nuclear volume and aspect ratios in embryos with both ground-truth and model-predicted segmentations. These comparisons revealed that the nuclear volumes and aspect ratios of the two matched well, particularly for the eight-cell stage up to the ≈80-cell stage (Figs S13 and S14). Therefore, for all nuclei in Fig. 5A, we used nuclear volumes and aspect ratios based on the corrected model predictions as proxies for the ground-truth quantities.

Since the nucleus volume relative to that of the cytoplasm (NC ratio) has been shown to impact cell cycles and gene expression in embryos of other species (Syed et al., 2021; Balachandra et al., 2022), we studied the dynamics of nuclear volumes from the eight-cell stage to the blastocyst stage. For preimplantation mouse embryos, the total embryo volume – excluding the cavity – is fixed because cell divisions simply partition existing volume (Aiken et al., 2004). By contrast, analysis of fixed embryos has revealed that nuclear volumes do not downscale as dramatically as cell volumes (Tsichlaki and FitzHarris, 2016). To quantify in a live embryo how these increases in NC ratio arise, we analyzed nuclear volumes across generations (e.g. from a mother at the 16-cell stage to two daughters at the 32-cell stage). Fig. 5B shows an ICM and a TE example of nuclear volume trajectories from the root of a lineage tree (eight-cell stage) to its leaves (≈100-cell stage) (see Fig. S12). After each division, the nuclear volume grew approximately linearly and reached a peak before the subsequent division (Fig. 5C; Fig. S12). To compare nuclear volumes at different developmental stages in a way that is not affected by asynchronous cell cycle progression, we used maximal nuclear volumes, measured for each cell immediately before mitosis.

We compared the maximal volumes of the daughter nuclei (*V_D_*_1,*max*_, and *V_D_*_2,*max*_) to that of their mother (*V_M,max_*) across developmental stages (Fig. 5C,D; Fig. S15B). If the nuclei were not downscaling with developmental stage at all, then *V_D_*_1,*max*_+*V_D_*_2,*max*_ would be expected to be ≈2*V_M,max_*. If the nuclei were downscaling to fix the NC ratio, we would expect *V_D_*_1,*max*_+*V_D_*_2,*max*_ to be ≈*V_M,max_*. For the 8-to-16 and 32-to-64 cell transitions, the sum of nuclear volumes of the two daughters (*V_D_*_1,*max*_+*V_D_*_2,*max*_) was greater than *V_M,max_*, but less than ≈2*V_M,max_*. Thus, the daughter nuclei were indeed downscaling, but were not simply halving the nuclear volume of the mother. By contrast, for the 32-to-64 cell stage, the sum of the daughter volumes approximately equaled that of the mother (Fig. 5C-D′). This suggests that nuclear growth is reduced at later developmental stages (Fig. S12). Future work is required to uncover the specific biological mechanisms controlling developmental-stage-dependent nuclear growth.

During the 8-to-16 and 16-to-32 cell transitions, a previous study reported that differences in cell volume – particularly between ICM and TE cells – arise (Niwayama et al., 2019). By comparing the volumes of daughter nuclei resulting from the same division (*V_D_*_1,*max*_ and *V_D_*_2,*max*_) across developmental stages (Fig. 5C,E,E′; Fig. S15C), we tested when nuclear volume differences emerge in the embryo. For the 8-to-16 and 16-to-32 cell transitions, many of the divisions had pronounced asymmetries (Fig. 5E; Fig. S15C). By contrast, for the 32-to-64 cell transition, the daughter nuclei had approximately equal maximal nuclear volumes (Fig. 5C,E). Since the nuclear volume asymmetries in earlier divisions often correspond to cases of two progeny of differing ICM/TE fate (Fig. S12), decreased asymmetries at the 32-to-64 cell transition may reflect the lack of mixed ICM/TE progeny in divisions after the 32-cell stage (Posfai et al., 2017).

To further demonstrate that biological insights can be gained with our pipeline, we probed the relationship between cell fate specification (ICM versus TE) and cell cycle durations. Previous studies identified differences between cell cycle durations of inner and outer cells during the 16-cell stage (MacQueen and Johnson, 1983; McDole et al., 2011). To determine whether such cell cycle differences persist during the 32-cell stage, we imaged two additional embryos with the H2B-miRFP720 reporter (Fig. S16), as well as two embryos with H2B-miRFP720 and a Cdx2-eGFP reporter (Fig. S17) (McDole and Zheng, 2012), a key TF of the TE (Strumpf et al., 2005). Using our pipeline, we constructed lineages for each movie and extracted nuclear Cdx2-eGFP for each nuclear instance in the lineage trees, where appropriate (Fig. 5F,F′; Fig. S18). We classified ICM and TE cells either based on nuclear position at the end of live imaging in H2B-miRFP720 embryos (Fig. 5G) or by averaging nuclear Cdx2-eGFP intensities during the 32-cell stage and classifying cells as Cdx2^−^ or Cdx2^+^ (Fig. 5H). Both these approaches revealed that Cdx2 high or TE-biased cells at the 16-cell stage indeed display shorter cell cycles compared to Cdx2 low or ICM-biased cells (Fig. 5I). Additionally, our analysis of the 32-cell stage showed that, although both ICM and TE cell cycles lengthened, the Cdx2 high or TE-biased cells similarly showed accelerated cell cycles compared to Cdx2 low or ICM-biased cells (Fig. 5I′). These data provide insight into how cell fate and cell cycle progression are dynamically coupled.

### Generalization of our trained Stardist-3D models to different experimental systems and a different imaging modality

Since previous studies have reported that deep neural networks can generalize well to unseen datasets (Hallou et al., 2021; Caicedo et al., 2019; Stringer et al., 2021), we tested whether our BlastoSPIM-trained models could generalize to other model systems and imaging modalities. In each generalization test below, we first evaluated our early embryo and late blastocyst models on the test set without any additional training. Then, we updated the weights of our early embryo and late blastocyst models by training on a small set of ground-truth data from the system of interest. We compared those so-called fine-tuned models to a model trained only on ground-truth data from the system of interest.

**Fig. 6.**
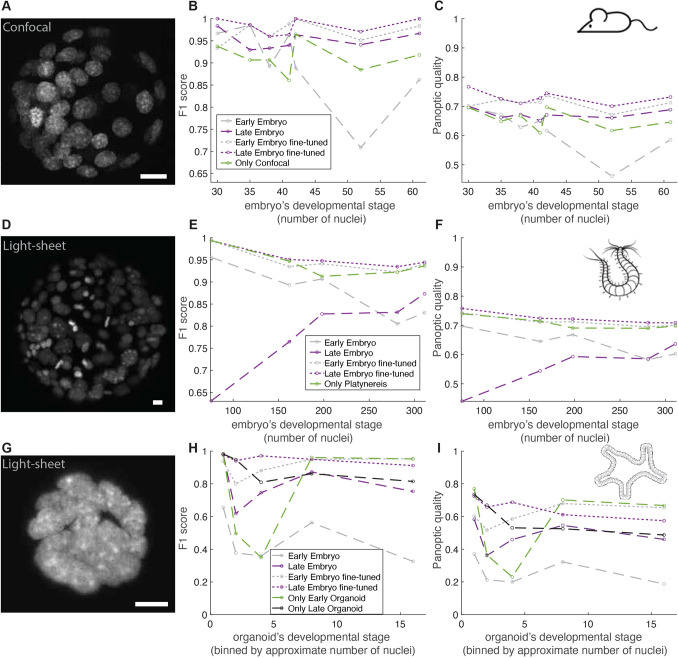
**Generalization tests for different model systems and a different imaging modality.** (A-C) For mouse embryos imaged via spinning-disk confocal microscopy, we generated ground-truth nuclear segmentation (see example maximum intensity projection in A). Panels in B and C indicate the performance of different models on the ground-truth test set. (D-F) Same as in A-C but for a ground-truth set of *Platynereis dumerilli* embryos (Lalit et al., 2022). (G-I) Same as in A-C but for a ground-truth set of intestinal organoids (De Medeiros et al., 2022). For each case, fine-tuning of our networks enables superior performance compared to the network(s) trained on the system-specific ground truth alone. Scale bars: 10 µm.

First, to test the generalization of BlastoSPIM-trained models on a different imaging modality, we generated a ground-truth set of ten preimplantation mouse embryos from the ≈32-cell stage to the ≈64-cell stage on a spinning disk confocal microscope (Fig. 6A). We set aside two embryos for training and validation of a new ‘only confocal’ model. We used that same set to update the weights of our two BlastoSPIM-trained Stardist-3D models. On the remaining embryos, the late blastocyst model – with and without training on confocal data – outperformed the ‘only confocal’ model (Fig. 6B,C). The fine-tuned early embryo model also outperformed the ‘only confocal’ model. Thus, the late blastocyst model performed well out-of-the-box, and fine-tuning on a small training and validation set significantly improved the performance of both the early embryo model and the late blastocyst model. Thus, our models generalized well, alleviating the necessity of generating a large new ground-truth set of confocal data.

We quantified whether our models generalized to datasets from other model organisms. We evaluated our Stardist-3D models on a ground-truth set of live light-sheet images of *Platynereis dumerilli* embryos from the 38- to the 392-cell stage (Lalit et al., 2022) (Fig. 6D), in which nuclei were labeled by microinjection of a fluorescent tracer. Applying our ‘early embryo’ model to the five *P. dumerilli* images, we found that it performed well, at ⪆90% *F*_1_ score, on early *Platynereis* embryos, from the 76- to 198-cell stages. For later stages, the ‘late blastocyst’ model outperformed the ‘early embryo’ model (Fig. 6E). We, then, fine-tuned both of our Stardist-3D networks on a set of four *P. dumerilli* embryos. We used the same sets to train and validate a new network, which we call the ‘only *Platynereis*’ model. The fine-tuned ‘late blastocyst’ model outperformed the ‘only *Platynereis*’ model (Fig. 6E,F) across the test set, with the most pronounced improvement at the ≈200-cell embryo. For the three test images at late stages (with 198, 281, and 311 nuclei), the fine-tuned network generated ≈30 fewer errors, including false positives and false negatives, and increased the mean IoU for matched instances at each timepoint. Notably, our models generalized to images of *P. dumerilli* embryos despite several differences in this data compared to images of mouse embryos, such as highly variable nuclear sizes and different nuclear textures (dense heterochromatic foci).

Finally, we tested how generalizable our models are to another system, intestinal organoids (De Medeiros et al., 2022) (Fig. 6G). While our ‘early embryo’ and ‘late blastocyst’ models did not perform as accurately out-of-the-box as they did on the previous two sets, fine-tuning of both of these models on sets of images of ten organoids with ground truth significantly improved their performance (Fig. 6H,I). For example, the fine-tuning of the early embryo model on early organoid data (<14 nuclei) and of the late blastocyst model on late organoid data (≥14 nuclei) allowed these to outperform the ‘only early organoid’ and ‘only late organoid’ models (trained on early and late organoid data, respectively). Surprisingly, the best improvement in performance relative to the ‘only organoid’ models occurred at the 2- to 4-cell stages (Fig. 6H,I). At these stages, a lumen has not yet formed or is small, and the large nuclei of the cells are closely juxtaposed and flattened. The large training set of blastocyst embryos (in BlastoSPIM 1.0 and 2.0) enabled our models to avoid merging of these nuclei in the segmentation.

Thus, our BlastoSPIM-trained models, either ‘out-of-the-box’ or fine-tuned with minimal ground truth from another system, can greatly improve nuclear segmentation accuracy in different types of imaging data (Fig. 6). We include code for training our BlastoSPIM models further on other ground-truth datasets (blastospim.flatironinstitute.org). With these new models used for segmentation, the pipeline (Fig. 4) can be used to segment and track nuclei in other related contexts.

## DISCUSSION

To understand how the behaviors of individual cells contribute to morphogenetic events, biologists acquire staggering amounts of time-lapse images. Quantifying the properties and behaviors of cells in such image series requires instance segmentation: identifying which voxels belong to which object. Although many measurements require segmentation of entire cells, instance segmentation of nuclei is useful for estimating the relative positions of cells, classifying by mitotic stage, and measuring the expression of nuclear-localized factors. Nuclear instance segmentation is challenging for several reasons, including nucleus-to-nucleus proximity, variations in nuclear shape, and voxel anisotropy. Since the application of supervised machine learning methods to instance segmentation often requires large ground-truth datasets, here we generated a publicly available ground-truth dataset, called BlastoSPIM, which is the largest 3D dataset of nuclear instance segmentation ground truth of its kind. Such large, fully annotated datasets are extremely useful resources, including for the benchmarking of different methods. Here, by comparative analysis of seven different neural networks on this new dataset, we have shown which of these networks best addresses the challenges of nuclear segmentation in the preimplantation mouse embryo (Fig. 2; Figs S5 and S6).

Our comparative analysis revealed state-of-the-art performance by Stardist-3D (early embryo model) across developmental stages. From the eight-cell stage up to the 64-cell stage, Stardist-3D's *F*_1_ score remained above 95%, and its panoptic quality at ≈80% (Fig. 2A). In contrast, the performance of other methods varied, with Cellpose and RDCNet producing many false positives, particularly at early developmental stages, and U3D-BCD and UNETR merging several nuclei for the 64-cell stage and later stages (Fig. 2). To further improve segmentation performance at later stages of preimplantation development, we hand-annotated a second ground-truth dataset of nuclei in late blastocyst embryos and trained a second Stardist-3D model (late blastocyst model), for which the *F*_1_ score remained above 90% for the >100-cell stage embryos (Fig. 3A). Therefore, we not only present trained Stardist-3D models with superior performance for nuclear instance segmentation in time-lapse images of early mouse embryos, but also share large ground-truth datasets (BlastoSPIM 1.0 and 2.0), which will be an important resource for evaluating the performance of future methods because of the dataset size and quality and nuclear density relative to other currently available datasets (Table S2).

Owing to our interest in studying preimplantation mouse development by live imaging, we integrated our Stardist-3D models into a complete image analysis pipeline (Fig. 4), including time-lapse acquisition, instance segmentation, segmentation correction, embryo registration, lineage tracking, and signal extraction. The models and the code underlying the pipeline are publicly available at blastospim.flatironinstitute.org. We used this pipeline to analyze time-lapse images of five different embryos from the eight-cell stage to at least the 64-cell stage, with one embryo analyzed up to ≈100-cell stage (Fig. 5). These segmentations revealed oscillations of nuclear volume with the cell cycle: volumes gradually increased throughout interphase and peaked just before mitosis, resulting in a sudden volume drop. We probed how nuclear volumes downscale from earlier developmental stages to later stages. Whereas previous studies have quantified this effect based on fixed embryos or live images without tracking, we used our lineage trees to compare the maximum volume of each mother nucleus to the maximum volumes of the resulting daughter nuclei (Fig. 5C,D; Fig. S15B). We found that nuclei did indeed downscale in volume over time, but that the maximum nuclear volumes of the daughter were not simply half that of the mother (see Fig. S15 for ground-truth examples). Additionally, sibling nuclei often differed significantly in nuclear volume at early developmental stages (Fig. 5C,E; Fig. S15C), but such asymmetries decreased considerably by the 32-to-64 cell transition (Fig. 5C,E). Furthermore, for two embryos expressing both H2B-miRFP720 and Cdx2-eGFP (McDole and Zheng, 2012), we extract Cdx2 intensities for each nucleus in the lineage trees (Fig. 5F,F′; Fig. S18) and examine how the dynamics of ICM/TE fate specification are correlated with cell cycle durations (Fig. 5I,I′). This analysis demonstrates that the pipeline can be used to analyze the dynamics of fate specification in multi-channel time-lapse images. We expect that our instance segmentation models will enable many insights into the development of preimplantation mouse embryos.

The usefulness of datasets like BlastoSPIM often extends to images acquired by different modalities or of different model systems. Here we tested whether: (1) models trained on BlastoSPIM can be applied off-the-shelf to segment nuclei in other contexts and (2) BlastoSPIM can be used to improve accuracy in segmentation via pre-training when limited ground-truth data is available. Our generalization tests extended across three datasets: preimplantation mouse embryos imaged by spinning-disk confocal microscopy, intestinal organoids imaged by light-sheet microscopy (De Medeiros et al., 2022) and *P. dumerilli* embryos imaged by light-sheet microscopy (Lalit et al., 2022). Our early embryo model and late blastocyst models worked well out-of-the-box for the spinning-disk confocal set and up to the 200-cell stage for the *P. dumerilli* embryos. Furthermore, fine-tuning of our models on the spinning-disk confocal set and the *P. dumerilli* embryos improved model performance relative to a model trained on system-specific data alone. Finally, fine-tuning of our models on the intestinal organoids set improved performance relative to an ‘organoid only’ model, particularly for stages when the nuclei were densely packed without a separating lumen. Thus, just as with other large ground-truth datasets (for example, ImageNet for object recognition in 2D images; Deng et al., 2009), finetuning or transfer learning from models trained on BlastoSPIM should improve performance on related tasks.

The generalizability of our model fills a clear need as many publicly available models work only in 2D or segment only cell boundaries (Koushki et al., 2023). Given the performance of our model even without fine-tuning, small hand-corrections of its predictions on a different biological system could be used to generate training data, as long as that system's nuclei can be approximated as star-convex polyhedra (Weigert et al., 2020; Schmidt et al., 2018). We expect that BlastoSPIM and our Stardist-3D models, in conjunction with other datasets and pre-trained models (Ouyang et al., 2022 preprint; Vijayan et al., 2024), will play a key role in the development of truly generalist models. Furthermore, the integration of BlastoSPIM-trained models into a larger analysis pipeline enabled the construction of lineage trees, which revealed the temporal dynamics of individual nuclei – in volume, shape, and TF intensities – as fate decisions transpire. This work is thus a crucial step towards fully automated 4D analysis of early mouse development, and modest modifications of our pipeline (Fig. 4) should enable analysis of early embryonic development in time-lapse images of other model systems.

## MATERIALS AND METHODS

### Transgenic mouse lines

The H2B-miRFP720 transgenic mouse line was generated by targeting the TIGRE locus using the 2C-HR-CRISPR method (Gu et al., 2018). Two targeting plasmids were constructed with InFusion cloning (Takara Bio): one consisted of 5′ and 3′ homology arms (each 1 kb in length), surrounding H2B-miRFP720 driven by a CAG promoter and flanked by rabbit β-globin polyA sequence; the second construct contained an additional ORF-2A preceding H2B-miRFP720 flanked by a bGH polyA sequence. A single guide RNA (sgRNA) designed using CRISPOR (Concordet and Haeussler, 2018) was used to target the TIGRE locus: CAUCCCAAAGUUAGGUGUUA (Synthego). CD1-IGS mice (Charles River, strain 022) were used as embryo donors. Briefly, female CD1-IGS were super ovulated at 5-7 weeks of age using 7.5 IU PMSG (Biovendor) administered by intraperitoneal (IP) injection followed by 7.5 IU HCG (Sigma-Aldrich) by IP injection 47 h post PMSG. Super ovulated females were mated to CD1-IGS stud males (8-30 weeks of age) and checked for copulatory plug the following morning.

Cytoplasmic microinjection of two-cell embryos was performed as previously described (Gu et al., 2020, 2018). Briefly, embryos were harvested at the two-cell stage on E1.5 by flushing the oviducts with M2 Media (Cytospring) and each cell was microinjected with 100 ng/µl Cas9 mRNA [made by IVT (mMESSAGE mMACHINE SP6 transcription kit, Thermo Fisher Scientific) using Addgene plasmid #122948], 30 ng/µl donor plasmid and 50 ng/µl sgRNA, using a Leica Dmi8 inverted epifluorescent microscope, an Eppendorf Femtojet and a Micro-ePore (WPI). Embryos were immediately transferred into the oviducts of pseudopregnant female CD1 mice. N0 pups were identified using over-the-arm PCR primers (Fwd: tcagcctacctcaccaactg; Rev: ccccatcgctgcacaaaata) and outcrossed to CD1-IGS mice. N1 animals were genotyped using the same primers and the transgene was Sanger sequenced. The N1 generation was further outcrossed twice before incrossing the line to obtain homozygous mice. Homozygous and heterozygous offspring were distinguished using a PCR of the TIGRE locus (TIGRE Fwd: CTTTCCAGTGCTTCCCCAAC; TIGRE Rev: CCCTTTCCCAAGTCATCCCT).

The first mouse line showed decreasing levels of H2B-miRFP720 fluorescence during preimplantation development, while the second ORF-2A-H2B-miRFP720 mouse line showed ubiquitous high expression throughout. Therefore, the ORF sequence was deleted in two-cell embryos isolated from this mouse line using the following sgRNAs: GGUGACGCGGCGCUGCUCCA and CAUGCCCAUUACGUCGGUAA. This resulted in a truncated ORF with a functional 2A peptide. Founders and subsequent generations were established from this line, herein referred to as the H2B-miRFP720 mouse line, and ubiquitous H2B-miRFP720 fluorescence was confirmed once again in embryos. See the Data availability statement for the link to the sequence of the H2B-miRFP720 transgene. Other transgenic mouse strains used in this study include Cdx2-eGFP (McDole and Zheng, 2012), mT/mG (Muzumdar et al., 2007), and YAP-emiRFP670 (Gu et al., 2022). Mice were housed in an Association for Assessment and Accreditation of Laboratory Animal Care International-accredited facility following the Guide for the Care and Use of Laboratory Animals. Animal maintenance and husbandry followed the laboratory Animal Welfare Act. The Princeton University Institutional Animal Care and Use Committee approved all animal procedures (IACUC protocol number 2133).

### Whole-mount immunofluorescence of preimplantation embryos

Embryos were obtained at E2.5 (eight-cell stage) from CD1 females crossed to H2B heterozygous males and cultured or imaged in KSOM until E4.5. Embryos were then fixed in 4% formaldehyde at room temperature for 10 min, the zona pellucida removed with acid tyrode (Sigma-Aldrich), permeabilized for 15 min in PBS 0.2% Triton X-100 and blocked in PBS containing 0.1% Tween-20 (PBS-T) with 2% bovine serum albumin (Sigma-Aldrich) and 5% normal donkey serum (Jackson ImmunoResearch) at room temperature for 1 h. Embryos were incubated in primary antibodies diluted in blocking solution overnight at 4°C, washed with PBS-T and incubated in secondary antibodies diluted in blocking solution for 1 h at room temperature. Embryos were washed three times, with the last wash containing Hoechst dye 33342 (Promega). Embryos were imaged in PBS on a confocal microscope. Primary antibodies used were: mouse anti-Nanog (BD Biosciences, 560259, RRID:AB_1645261, clone: M55-312, lot: 8250916, 1:200), goat anti-Sox17 (R&D Systems, AF1924, RRID:AB_355060, batch: KGA1223081, 1:100). Secondary antibodies used were: donkey anti-mouse AlexaFluor555 (Abcam, ab150106, RRID:AB_2857373, batch: GR3220544-2, 1:500), donkey anti-goat AlexaFluor488 (Thermo Fisher Scientific, A-11055, RRID:AB_2534102, batch: 2059218, 1:500).

### Quantification of fixed and live imaged embryos

Embryos were genotyped by presence of H2B-miRFP720 signal. For each embryo, the number of Nanog^+^ and Sox17^+^ cells were counted manually. PE cells were classified as Sox17^+^, regardless of Nanog expression. EPI cells were classified as only positive for Nanog. Total cell number was obtained by segmenting confocal images on the Hoechst channel, hand correcting the ROIs and counting the number of unique labels. Any embryos that did not develop to the 64-cell stage or beyond were excluded. We note that a similar proportion of embryos did not develop beyond the 64-cell stage in each experimental group. TE cell number was obtained by subtracting any Nanog/Sox17-expressing cells (ICM) from the total cell number. For comparison to live imaged embryos, the final timepoint was segmented on the H2B-miRFP720 signal, ROIs were hand corrected and the number of unique labels were counted. TE and ICM cells were identified by position from this image. We estimated any effects of the genetic modification (H2B-miRFP720) and of the imaging condition by conducting two main statistical comparisons: (1) incubator WT to incubator H2B, (2) unimaged H2B to imaged H2B. These comparisons, via a Wilcoxon rank-sum test, were on the basis of total cell number. We additionally show that three other measurements (the proportions of PE, EPI, and TE) are comparable between incubator WT and incubator H2B and between unimaged H2B and imaged H2B. We summarize the data in Fig. S2.

### Dataset acquisition

Embryos were obtained from naturally mated or super ovulated H2B-miRFP720 females mated to either WT (CD1) or H2B-miRFP720 males. For the demonstration of four-color imaging, *YAP-emiRFP670; Cdx2-eGFP* females were mated to *H2B-miRFP720; mT/mG* males. Embryos were isolated at E1.5 (two-cell) or E2.5 (eight-cell) in M2 media and were cultured in Embryomax KSOM (Sigma-Aldrich) under paraffin oil (Life Global Paraffin Oil, LGPO, Cooper Surgical) in a V-shaped imaging chamber at 37°C, with 5% O_2_ and 5% CO_2_. Images were acquired on an InVi SPIM (Luxendo/Bruker). For each fluorescent reporter, the following excitation lasers and emission filters were used: eGFP 488 nm laser, 497-554 BP filter; tdTomato 561 nm laser, 577-612 BP filter; emiRFP670 642 nm laser, 659-690 BP filter; miRFP720 685 nm laser, 700 LP filter. To limit light exposure to the embryo, we acquired a full 3D image of each embryo at 15-minute intervals, with 2.0 µm *z*-axis resolution and 0.208 µm *x*- and *y*-axis resolution. Typically, the embryos were imaged from the eight-cell stage until the 64-cell stage or to the >100-cell stage, resulting in a duration of 48 h or more. Raw time-lapse images were compressed to keller-lab-block (klb) format, on the fly. Blastocyst images used in Fig. 6A-C were acquired using a W1 spinning disk confocal with SoRa on an inverted Nikon Ti2 with Hamamatsu ORCA-FusionBT, with a 20× (NA=0.75) air objective.

### Dataset annotation

Raw 3D images of developing embryos were manually annotated using AnnotatorJ, an ImageJ plugin that supports both semantic and instance annotation. Images were loaded into the tool as *z*-stacks in .tiff format. For all images, brightness and contrast were adjusted by using the ‘auto’ and ‘reset’ functions in ImageJ. ‘Instance’ was selected as the annotation type. For each nucleus, the top or the bottom slice was found by comparing consecutive *z*-slices, and a contour was drawn for every slice that contained the nucleus. The coordinates of the ROIs enclosed by the contours were then saved in an individual file. After each instance was annotated, the contours were overlaid on the image to distinguish the instance from unannotated ones. Five individuals annotated the images, and an expert checked for annotation errors via a custom MATLAB code, before incorporation into the dataset.

Annotation of chromatin signal in mitotic cells, particularly those in metaphase and anaphase, presents unique challenges. Because our nuclear reporter H2B-miRFP720 is a tagged histone, chromatin condensation makes the fluorescent signal bright and often irregularly shaped. During metaphase, the ‘nuclear’ instance was annotated as contiguous, and its contour in each *z*-slice was drawn to closely match the shape of the signal. In anaphase and telophase, two instances (with distinct instance labels) were drawn and made to conform closely with the boundary of the bright condensed signal. We carefully annotated the H2B signal in metaphase, anaphase and telophase because it is important for the trained network to segment these nuclei well: the shape – particularly the orientation of the metaphase plate – and volume can be particularly informative for the assignment of daughter nuclei to mother nuclei in time lapse images (see Materials and Methods, Nuclear tracking).

### Dataset characteristics

The BlastoSPIM 1.0 dataset includes 573 fully annotated 3D images of nuclei in mouse embryos, each manually curated for annotation. Across all images, there are 11,708 individual nuclear instances annotated and 116 annotated polar bodies. Not all of these 3D images come from different time series. For example, for one developing mouse embryo, we annotated 89 consecutive timepoints, and for another embryo, we annotated 100 consecutive timepoints. The total number of distinct embryos imaged and annotated is 31.

Aside from diversity in developmental stage, the embryos in this dataset express different H2B-miRFP720 alleles (see Materials and Methods, Transgenic mouse lines) and were also imaged with different laser intensities. This diversity in laser intensities allows us to test whether model performance degrades significantly as the intensity of the nuclei (the foreground) decreases. To characterize these differences in laser intensities across images, we calculated the foreground-background intensity difference: the mean foreground intensity minus the mean background intensities. We report the distribution of this foreground-background intensity difference, one point for each fully annotated 3D image, in Fig. S3. For comparison, the background intensity – in gray values, which represents fluorescent intensity values at each voxel in the image – typically has a mean of 118 and a variance of 10-14. We define ‘low foreground-background intensity difference’ images for BlastoSPIM 1.0 as having a mean foreground intensity which is at most 134 gray values, approximately 15 gray values above the typical mean background intensity. For reference, the background intensity – in gray values – typically has a variance of 10-14 (Fig. S3). Every image that is not in the ‘low foreground-background intensity difference’ set is in the ‘moderate foreground-background intensity difference’ set.

The BlastoSPIM 2.0 dataset consists of 80 annotated images of late-stage embryos. This set includes 6628 nuclear instances. Late blastocysts from this dataset with the lowest foreground-background intensity difference values (Fig. S3) were selected and incorporated into the existing ‘low foreground-background intensity difference’ set. The final number of annotated images in BlastoSPIM 1+2 is 653, and the number of annotated nuclear instances is 18,336.

In addition, to demonstrate that the models trained on BlastoSPIM perform well also for images acquired via other modalities, we annotated the nuclei in ten different embryos imaged on a spinning disk confocal microscope (Fig. 6A-C). These ten images range from the 30-cell stage to the 61-cell stage and contain 461 nuclei in total.

### Dataset splits and evaluation metric

When splitting our dataset into a training set, a test set, and a validation set, our main objective was to quantify how model performance varies as a function of both developmental stage and foreground-background intensity difference. For BlastoSPIM 1.0 we created two separate test sets, one for low foreground-background intensity difference and one for moderate foreground-background intensity difference (see above), each of which contained a diversity of developmental stages. Within both the moderate foreground-background intensity difference and the low foreground-background intensity difference sets, we grouped annotated embryos based on their developmental stage, estimated by the number of nuclei (i.e. ≈8 cell, ≈16 cell, ≈32 cell, ≈64 cell, >100-cell). Each set deliberately contains more images from earlier stages than later stages so that the total of number of nuclei per developmental stage is at least partially equalized across stages. From the BlastoSPIM 2.0 dataset, eight embryos from various stages were used as a test set, as early as the 48-cell stage and as late as the 107-cell stage. The remainder of the data, 72 annotated embryos, were either for validation or training. The exact breakdown is specified at blastospim.flatironinstitute.org.

To evaluate how well the models performed on the test sets, we computed the IoU between the model segmentation and the ground truth. We considered an instance in the model segmentation to match an instance in the ground truth if the IoU between the two was at least 0.5. We also provide how performance varies as a function of this IoU threshold (Figs S5, S6, S8, S9). We assign an IoU of zero to each unmatched ground-truth instance (i.e. below the IoU cutoff). To see how well the predictions fit the ground truth, we compute the panoptic quality (Hirling et al., 2023): 

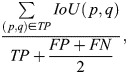
where (*p*,*q*) represents a match between predicted object *p* and ground-truth object *q*. *TP*, *FP*, and *FN* denote true positives, false positives, and false negatives, respectively. The train-test-validation split for all the models is specified on the BlastoSPIM website, blastospim.flatironinstitute.org.

### Segmentation correction methods

To achieve accurate lineage tracking, even infrequent segmentation errors need to be corrected. These errors include oversegmentation, undersegmentation, or misclassification of background noise as cells. To manually rectify these errors, we employed an enhanced version of the ImageJ plugin, AnnotatorJ (Hollandi et al., 2020).

AnnotatorJ provided users with an intuitive interface to inspect and identify segmentation errors by overlaying the segmented ROIs onto the original image. To cater to the specific needs of 3D image analysis, we introduced various enhancements to AnnotatorJ (v1.6). Briefly, AnnotatorJ v1.6 allowed for the display of ROIs in each frame of a 3D image, enabling users to navigate seamlessly through frames and address errors in each frame individually. This functionality was made possible by incorporating operations to add or remove regions, modify boundary positions, and correct misclassified areas manually. A ‘generate mask’ operation was added to easily generate the labeled mask image once corrections were completed. We implemented color coding of ROIs for easier identification and generation of a final corrected labeled mask image. Additionally, we introduced support for loading compressed KLB files, for efficient data handling.

### Registration methods

Under normal imaging conditions, the embryo can rotate and translate within the microscope field of view. While global positions of cells in consecutive frames may change, their relative positions are unlikely to change significantly. To achieve positional consistency over time, we use the coherent point drift (CPD) algorithm to register pairs of frames in sequence (Myronenko and Song, 2010). CPD is a point set registration algorithm which takes a probabilistic approach to aligning two sets of points. Each point set is represented by a Gaussian mixture model (GMM), and a transformation function is learned which maps the centroids of the first point set onto the centroids of the second set. For each pair of frames, we take the corresponding point sets to be the centroids of the distinct instances in the instance segmentation. We also restrict the transformation function to be a rotation and translation.

We found that CPD tended to converge to local minima on our data depending on the initial rotation guess. Because the point sets were small and thus the CPD execution time was low, the correct registration could be found quickly by running many CPD trials with randomly chosen initial rotations. The final registration was chosen as the trial which minimized the GMM covariance parameter. This covariance parameter acts as a length-scale which tends to be large for misaligned point sets and small for aligned point sets.

### Nuclear tracking

After the error correction and registration steps, we perform semi-automated lineage tracking on the registered nuclear segments. Our tracking algorithm constructs the lineage tree sequentially, at every iteration matching nuclei to their predecessors in the previous time frame. We build our algorithm on the previously published effort (Strnad et al., 2016) that was based on nearest neighbor association between nuclear centroids at adjacent time frames and, in the case of mitosis, searching for pairs of daughters with minimal distance to the mother. However, we found that while this method can be successful in tracking nuclei during interphase, the slower frame rate of our samples is an obstacle to the success of the algorithm in correctly identifying division events. Therefore, we introduced additional steps comparing nuclear volumes for every matched pair of nuclei to prune incorrect tree edges and identify mother-daughter triples, with the option for manual correction. This approach takes advantage of the observed differences in nuclear volume between daughter nuclei and their mother due to splitting of condensed chromatin. More precisely, for every pair of frames, we perform several steps. In the steps below, regardless of whether a division event occurred between times *t* and *t*+1, we refer to nuclei at time *t* as mothers (or parents, interchangeably) and nuclei at time *t*+1 as daughters. Only if a mother at time *t* has two daughter nuclei at *t*+1 has a division event occurred in the tree.

Step 1. We start by matching every label at time *t*+1 with its nearest neighbor at time *t* (using Euclidean distances between centroids), thus identifying prospective parents for every label. The matches can be viewed as the edges that are added to the tree at height *t*. For these initial edges, we compare the volumes of the two matched nuclei and first retain the edges that represent one-to-one mappings without significant volume disbalance (defined by the daughter-mother volume ratio larger than a user-defined threshold; heuristically, we set the default value to be 2/3). The rest of the edges represent many-to-one daughter-mother mappings. For every prospective mother, we identify how many of the prospective daughter nuclei are large enough, i.e., do not demonstrate significant volume disbalance to the mother (defined above). If there is just one large daughter identified, we retain this connection pruning all the rest; otherwise, we prune all the edges for this prospective mother. Thus, all the retained matches potentially represent the same nucleus which slightly changed its position between times *t* and *t*+1.

Step 2. At this point, the nuclei at times *t* and *t*+1 that belong to the edges retained at Step 1 are removed from consideration. The remaining nuclei at time *t*+1 undergo the next round of nearest neighbor mapping to time *t*. At this stage, one-to-one mappings are retained. Now we aim to identify for mitotic triples. To do this, we search for prospective parents (time *t*) that were mapped to exactly two nuclei at time *t*+1. We retain both edges for such a triple if there is significant daughter-mother volume disbalance (see definition in Step 1) for both prospective daughters. The mitotic triple criterion includes an option to check for the centroid of the daughters to be close enough to the prospective parent.

Step 3. All the remaining connections from Step 2 represent the many-to-one mappings that do not satisfy the criterion for the mitotic triple. We attempt to resolve such conflicting matches by using second nearest neighbors. If this procedure does not identify a plausible mitotic triple based on the criterion described in Step 1, we manually identify the correct mitotic triple and edit the lineage tree by using the rmedge and addedge functions in MATLAB.

### Effect of frame rate on tracking

In this study, we chose 15 min as the time step between subsequent frames in the time lapse. This is longer than the time step used in some previous studies (Strnad et al., 2016; McDole et al., 2011), which only generated images with one or two channels. Our longer time step allows us to acquire up to four channels, while minimizing the embryos' exposure to light. We demonstrate, particularly in Fig. 5A, that our coarser time resolution is sufficient for tracking nuclei from the eight-cell stage to the late blastocyst stage. We note two main challenges for tracking nuclei across time: mitoses and embryo rotation. Since the duration of cytokinesis is ≈10 min for the stages of development from eight-cell to late blastocyst (Paim and FitzHarris, 2022), any time step much greater than 10 min will generate time lapses where multiple nuclei divide between consecutive frames, thus impeding the reliable assignment of daughter nuclei to mother nuclei at the time of division. On the other hand, as long as the number of mitotic events between frames remains low, our registration code effectively corrects for embryo rotations. Of note, increasing frame rates does not necessarily make the process of registration easier as the embryo can complete large rotations on the timescale of ≈1 min.

### Extraction of nuclear TF intensity

To analyze Cdx2-eGFP signal from time-lapse images of mouse embryos expressing both H2B-miRFP720 and Cdx2-eGFP (McDole and Zheng, 2012), we have developed a pipeline for TF nuclear intensity extraction. As input, the pipeline takes nuclear segmentation (provided by Stardist-3D) and the corresponding TF intensities in 4D captured in the same embryo in the same imaging session. The pipeline independently averages TF intensity over every nuclear segment at every individual timepoint and outputs the values in a csv format. Since the histone channel and the TF of interest might be captured by different cameras, the pipeline includes an optional camera alignment step which precedes the extraction. To align the cameras, we search for a translation of the TF intensity 3D image in the *xy* plane that maximizes the average intensity inside the corresponding nuclear segments. This is done for a timepoint pre-defined by the user and brute-force optimization over a large pre-defined grid of *xy* translation vectors.

### Analysis of nuclear TF intensity

To correlate cell cycle lengths with Cdx2-eGFP behavior, we extracted Cdx2-eGFP intensities in two collected embryos and classified branches in those embryos as Cdx2^−^ or Cdx2^+^. For this classification, we only considered cells until their 32-to-64 cell stage division and thresholded on their mean Cdx2-eGFP intensity *c_i_* at the 32-cell stage. In one of the embryos, we excluded two 32-cell stage sister cells from consideration as outliers due to the abnormally long (23.75 h) 16-cell stage duration of their mother. As a result, 62 cells (branches) were initially considered for classification. Owing to differences in average intensities of Cdx2-eGFP in two embryos that we collected, we first standardized the extracted signal dividing by the mean of all observed *c_i_* values for the embryo. We then used a kernel density estimate (kdeplot method from seaborn python package with default parameters) for the observed 62 normalized values that produced a bimodal distribution. To define the threshold of Cdx2-eGFP intensity, we searched for a local minimum of the density that allowed us to separate two modes. Cells with *c_i_* below the threshold were classified as Cdx2^−^, the rest of the cells were classified as Cdx2^+^. Only cells with complete 16- and 32-cell stage cycles were considered to produce the plots in Fig. 5I,I′. The assignments could then be propagated along the corresponding branch, e.g., to correlate 16-cell stage cell cycle lengths with future class assignments.

### Construction of the lineage trees from the eight-cell stage to the >64-cell stage

For Fig. 5A, the initial segmentation was produced using the ‘early embryo’ Stardist-3D model for frames 1 through 145, at which point the embryo cell count reached 64. For the remainder of the movie, ending at frame 210, we switched to the ‘late blastocyst’ Stardist-3D model as it achieves a higher accuracy in later stages and reduces the number of error corrections needed. Following segmentation, we used our enhanced AnnotatorJ tool to hand-correct errors and began tracking using our semi-automatic lineage construction algorithm. The process of lineage construction provides temporal context that often reveals errors in the segmentation that may be non-obvious when viewing a segmented frame in isolation. For this reason, performing hand-correction and lineage construction in parallel is useful. After building the lineage tree, ICM and TE fates were assigned based on nuclear shape (TE nuclei are typically more flat) and position (TE nuclei are closer to the surface, whereas ICM nuclei are positioned deeper in the embryo).

From the beginning of the 32-cell stage to the 64-cell stage, the estimated volume of each nucleus was computed by averaging the predictions of the early and late models. More specifically, since only one of these two models was corrected per time point for the purpose of the lineage tree, we computed matches between the corrected segmentation (from either the early or late model) and an uncorrected segmentation from the other model. If a match of sufficiently high IoU was identified, then the volumes of the nuclear instance in the two models were averaged. If no sufficiently matching instance was found, then only the volume of the instance in the corrected segmentation was used. Fig. S13 demonstrates that the model-predicted instances have volumes which agree reasonably well with those of the ground-truth matches; thus, for Fig. 5 we used model-predicted nuclear volumes as a proxy for the ground-truth volumes (which can only be obtained by full manual annotation – not done for these lineage trees).

The four other embryos with lineage trees reaching the ≈64-cell stage or beyond (the data underlying Fig. 5F,F′,I,I′) were produced in the same way, by transitioning from the early embryo model to the late blastocyst model at the stage determined by our model evaluations (Fig. 3A,B) and correcting the nuclear segmentation for lineage construction.

### Quantifying nuclear volume and aspect ratio

For the quantification of nuclear aspect ratio, we use the procedure – based on calculating the moment of inertia tensor – described in Niwayama et al. (2019). The quantification of volume was obtained by multiplying the number of voxels in the instance with voxel volume. For Fig. 5D,E, to reduce noise in estimated nuclear volume, each maximal volume (*V_max_*) per cell stage (8-to-16, 16-to-32 or 32-to-64) was calculated by averaging the nuclear volume for 90 min preceding the nuclear volume peak (see Fig. 5B).

For calculating maximal nuclear volumes in Fig. 5D,E, the 8-to-16 and 16-to-32 divisions are unambiguously defined because they occur in two temporally distinct rounds. On the other hand, because cell cycle asynchrony increases with developmental time, the definition of a 32-to-64 cell division is potentially ambiguous: namely, some 16-to-32 divisions have not happened even as some daughters of other 16-to-32 divisions have themselves divided (see Fig. 5A). For each tree, along a path from the root to the leaves, we define the 32-to-64 cell divisions as those which – without any intervening divisions along the lineage path – follow a 16-to-32 division. For the purposes of Fig. 5D,E, we only plot the 32-to-64 divisions that result in two progeny which both survive until the end of tree and divide at least once more. We make this choice because those are the only cases in which the quantities *V_D_*_1*,max*_+*V_D_*_2*,max*_ and *V_D_*_1*,max*_−*V_D_*_2*,max*_ are well-defined.

### Evaluating our tracking method in comparison to Trackmate

We evaluated the lineage trees produced by our technique and by Trackmate (Tinevez et al., 2017) against the hand-corrected lineage. To evaluate Trackmate, we provided as input either the registered or unregistered segmentation (Trackmate registered and Trackmate unregistered in Table S5). In Trackmate, we used the linear assignment problem (LAP) tracker with an allowance for track splitting. Trackmate uses two parameters, the frame-to-frame maximum distance and the lineage splitting maximum distance. We used a grid search to choose the best combination of both parameters. In the case of the unregistered segmentation input, we varied the frame-to-frame maximum distance and the lineage splitting maximum distance between 30 px and 90 px. For the registered segmentation input, we varied both parameters between 15 px and 60 px. The parameter sets identified as optimal (in this range) for Trackmate unregistered and Trackmate registered in Table S5 are: frame-to-frame distance=30 px, splitting distance=90 px and frame-to-frame distance=15 px, splitting distance=60 px, respectively.

### Network implementation details

Each model was trained with data from 482 3D images of whole embryos. Each embryo was cropped into 8-16 patches depending on the size for a total of 4363 patches. Each patch had a resolution of 64×256×256. The patches overlap such that all voxels of a nucleus were fully contained in at least one patch. The raw intensity images were bit-shifted by four bits to the right, so that all voxel intensities are in the range between 0 and 255. Any value still above 255 was capped at 255.

#### RDC Net

For all hyperparameter combinations sampled for training, a few were held constant. The downsampling factors were chosen to be 1, 10, and 10, for the *z*, *x*, and *y* directions, respectively, to account for anisotropy. Spatial dropout was chosen to be 0.1, following the original paper. All networks were trained for a maximum of 200 epochs, batch size of 2, with the Adam optimizer and Cosine Decay Restarts scheduler with learning rates from 10-3 to 0. The set of model weights that resulted in the lowest validation loss across all epochs was saved. The patch size was either the original crop (64×256×256) or 32×256×256 (32 random, consecutive *z*-slices from the original crop). The number of groups (parallel stacked, dilated convolution blocks with shared weights), dilation rates, number of channels per group, number of iterations, and the margin parameter were also adjusted to observe their effects on network performance.

During inference on test images, each raw image was broken into patches with the same size as those the model was trained on. The test patches were passed through the model and the resulting label patches were stitched together by discarding redundant masks and any masks touching the patch boundaries, assuming each nucleus is located at the center of at least one patch.

#### Cellpose

Since the original cellpose model is 2D, the 3D patches were broken into 2D slices for training. The source code was modified to include a data loader, since the size of the 2D training set is orders of magnitude larger than the original Cellpose dataset. Models were trained for a maximum of 1000 epochs, either from scratch or from a pre-trained Cellpose model. Test images were downsampled by a factor of 0.5 in *x* and *y* to improve performance since Cellpose is prone to oversegmentation for our full-resolution images in 3D. The patching and stitching method was the same as for RDCNet.

#### Stardist

3D Stardist was trained with patches of 32×256×256 sampled from the full-size patches. Input intensity was normalized capping values below 1% and above 99.8%. For sampling the star convex in 3D, we used 96 rays with a grid of 1×4×4 to compensate for the anisotropy. Data augmentation included 2D flips and grid warping.

#### U3D BCD and UNETR

The UNETR encoder transformer uses an embedding dimension of 768, the input volume is patched into volumetric tokens of dimensions 16×16×16, and multi-head self-attention is performed with 12 heads. Augmentations, in the form of randomized brightness and contrast, flips, rotations and elastic deformations, were used. Finally, the input volumes were randomly cropped to 16×128×128, before passing them through the network. Adam optimizer with decaying learning rate was chosen for training. Weighted sum of binary cross entropy (BCE) loss and dice loss is taken for foreground and contour masks, while mean squared error (MSE) was used for signed distance transform map predictions. Inference is performed by processing overlapping sliding windows across the large volumes of testing set. During post-processing, the multi-channel outputs from networks are combined by thresholding them appropriately to find instance seeds (or markers). Similarly, a more relaxed threshold on the outputs is used to obtain the foreground mask. Thereafter, marker-controlled watershed algorithm can be used with the help of seeds and predicted distance map to find instances.

#### QCANet

For QCANet, the original ground-truth set (BlastoSPIM 1.0) had to be converted to a set of nuclear centroids and semantic segmentation. The nuclear centroid image was computed by first downsampling the *xy*-resolution by 4 and then by setting the value of any voxels within 2.5 µm of a nuclear centroid to 1. The semantic segmentation was computed by setting all labeled regions in the instance segmentation to 1 and downsampling by 4 (using scikit-image: block reduce based on maxima). The instance segmentation was downsampled in the same way, for comparing model predictions to ground truth. In all cases, the downsampled ground truth always had the same dimensions: 64×173×169.

Images were read in as unsigned 16-bit tiffs, with the *z*-resolution ∼2.5× less than the *xy*-resolution (as done in the original QCA study). To handle the very bright polar bodies in our images, we changed the QCANet image normalization. Input intensity was normalized capping values below 1% and above 99.8% and then normalized using the CSBDeep normalize function. Both the training and validation sizes for both nsn and ndn networks were 5, with augmentation. For all training, the epoch number was set to 200.

#### ELEPHANT

To train a 3D ELEPHANT model, we converted our ground-truth data into ellipsoid labels, using the script generate_seg_labels.py in their latest (and continuously updated) main ELEPHANT repository (https://github.com/elephant-track/elephant-server). In the label generation step, the center ratio was set to 0.7. In the training step, the script train.py was used. We trained a model extending the pre-trained versatile model with our dataset of 64×256×256 crops using an additional crop size of 24×192×192, a batch size of ten, and for 40 epochs. We used the class weights for the negative log-likelihood loss (*class_weights_*): nucleus center=200, nucleus periphery=100, background=1, with a learning rate (lr) of 0.005. The validation dataset was built as a subset of the dataset by picking up every ten image/label pairs. The final model was selected based on the validation set and was from the last epoch (i.e. epoch 40). The training was carried out in ∼7.6 h. In the inference step, the script elephant-detection.py was used with the following parameters: scales of 2.0×0.208×0.208 µm, a patch size of 24×256×256, a batch size of 10, a minimum radius (*r_min_*) of 2 µm, a maximum radius (*r_max_*) of 10 µm, a center ratio (*c_ratio_*) of 0.7, and a probability threshold (*p_thresh_*) of 0.5.

#### Training with synthetic data

To counter the limited number of samples with densely-packed nuclei, we generate artificial samples to learn generalized features. This is made possible by modeling nuclei as 3D Gaussian kernels, of dimensions x, y, z where x, y∈(100, 150] and z∈(3, 6]. Elastic deformations, randomized lighting, and addition of noise are done to match SNR ratios with those of the actual dataset. The U3D BCD and UNETR models are pre-trained with this simulated data, allowing the network to fine-tune its predictions on the actual dataset.

### Model generalization tests

#### Mouse blastocysts imaged by spinning disk-confocal

The voxel resolution for these images is the same as that of our BlastoSPIM datasets. The validation set for the fine-tuned models and for the ‘only confocal’ model is a single embryo with 50 nuclei, in 32×256×256 patches. The training set for the fine-tuned models and for the ‘only confocal’ model was is a single embryo with 58 nuclei, in 32×256×256 patches. All models were trained as described in the Stardist section of Materials and Methods, Network implementation details. The fine-tuned models and the ‘only confocal’ model were trained in the same way, with the only difference being in the weights of the network at the start of training. Further improvement of the fine-tuned model relative to the ‘only confocal’ model could possibly be obtained by lowering the learning rate or fixing some network weights during fine-tuning.

For the ‘early embryo’ model and ‘late blastocyst’ model results without fine-tuning, we combined the validation and training embryos described above (one embryo with 50 nuclei and another with 58 nuclei) to optimize only the non-maximum suppression (NMS) threshold and probability threshold (using the built-in optimize_thresholds) without tuning any weights in the network.

#### *Platynereis dumerilli* embryos

The voxel resolution for the *P. dumerilli* images is 0.406 µm in *xy*, compared to 0.208 µm for the BlastoSPIM datasets, while the *z* resolutions of both sets are equal (Lalit et al., 2022). We, thus, upsampled the images of *P. dumerilli* embryos by a factor of two in *xy* (by interpolation with rescale from sci-kit image). The validation set for the fine-tuned models and for the ‘only *Platynereis*’ model contains half of 32×256×256 patches of two images, one with 38 nuclei and the other with 392 nuclei. The rest of the 32×256×256 patches of these two images, in addition to 32×256×256 patches of images with 113 nuclei and 261 nuclei, are in the training set.

All models were trained as described in the Stardist section of Network Implementation Details. The fine-tuned and the ‘only confocal’ models were trained in the same way, with the only difference being in the weights of the network at the start of training. Further improvement of the fine-tuned model relative to the ‘only confocal’ model could possibly be obtained by lowering the learning rate or fixing some network weights during fine-tuning.

For the ‘early embryo’ model and ‘late blastocyst’ models results without fine-tuning, we combined the validation and training embryos described above to optimize the NMS threshold and probability threshold without tuning any weights in the network.

#### Intestinal organoids

The organoids dataset was provided by the Liberali Lab (see De Medeiros et al., 2022). Images were acquired using light-sheet recordings performed with a dual-illumination inverted light-sheet microscope. Images were acquired with an *xy* resolution of 0.26 µm and 2.0 µm between slices. The dataset contained three sequences of live intestinal organoids grown from single cells, two that were budding and one with a growing enterocyst. As compared to nuclear shapes in early preimplantation embryos, the nuclear shapes in intestinal organoids vary considerable across time and across different cells. Additionally, in images of intestinal organoids, the large nuclei are closely juxtaposed even before the formation of a lumen.

We used frames 21 to 350 of the first budding organoid (starting with two nuclei and reaching ≈40 nuclei) for training and frames 21 to 350 of the enterocyst (starting with one nucleus and reaching ≈30 nuclei) for testing. We used these frames because these images had label images which appeared to be the most accurate with respect to the raw data.

We were given the original raw images and the deconvolved images but found that the models performed better with the raw images. This is not surprising, since the BlastoSPIM-trained models were trained on raw images as well. Denoising and deconvolving did not improve network performance although they improve the visual appearance for human viewing.

### Computational resource requirements

For training of any of the models presented in this study, either a 40 GB A100 or a 32 GB V100 was used. Training was not parallelized across multiple GPUs. Although the use of a GPU tends to speed up the process of model inference, a GPU is not required for that step. For inference, we requested 150 GB memory on nodes with 2.6 GHz Intel Skylake CPUs. With 16 threads requested it takes on average 120-130 s per image. Additionally, for the use of our models on new data from different model systems or imaging modalities, the optimization of the NMS threshold and probability threshold – as opposed to the model weights themselves – with the use of the Stardist-3D function optimize_thresholds does not require a GPU.

## Supplementary Material

10.1242/develop.202817_sup1 Supplementary information
