# Supplementary material for: Nuclear instance segmentation and tracking for preimplantation mouse embryos
Source: Development. 2024 Nov 6;151(21):dev202817. doi: 10.1242/dev.202817 (PMC11574361; doi:10.1242/dev.202817)
Supplement: Supplementary information [file develop-151-202817-s1.pdf]

Supplementary Materials and Methods

Estimate of number of nuclear instances in time-lapse images

The total number of nuclear instances in our time-lapse images from the 8-cell stage to the late blastocyst (> 100-cell stage) stage makes full manual annotation of the instances impractical and thus reinforces the usefulness of our analysis pipeline. To approximate the total number of instances, we note that we typically image the last four hours of the 8-cell stage. We assume for simplicity that each subsequent round of divisions is synchronous. Based on previous studies (Ciemerych and Sicinski, 2005; Fabrèges et al., 2023), the cell cycle durations at the 8-cell stage and 16-cell stage are ≈ 12 hours - 14 hours. The mean (and median) inter-division time increase between subsequent cell cycles (Fabrèges et al., 2023); to reflect this, we assume the 16-, 32-, and 64- cell stages last for 13, 14, and 15 hours respectively. Since we image the embryo for a couple of hours following the 64-to-128-cell divisions. Summing these durations, we find that ≈ 48 hours of imaging is required; with our time step of 15 mins, there are ≈ 200 frames (16 at the 8-cell stage, 52 at the 16-cell stage, 56 at the 32-cell stage, 60 at the 64-cell stage, and 8 at the 128-cell stage). To approximate the number of nuclear instances, we sum the products of the number of instances at each stage (8,16,32,64,128) with the number of frames at each stage. This returns an estimate of ≈ 8000 instances. In this study, for the two embryos tracked to the late blastocyst (> 64 cell), we found 9251 nuclear instances in the tree in Figure 5(A) (from the 8-cell stage to the 98-cell stage) and 5351 nuclear instances in Fig. S16(B) (from the 8-cell stage to the 72-cell stage).

Development • Supplementary information

References

Bondarenko, V., Nikolaev, M., Kromm, D., Belousov, R., Wolny, A., Rezakhani, S., Hugger, J., Uhlmann, V., Hufnagel, L., Kreshuk, A. et al. (2022). Coordination between embryo growth and trophoblast migration upon implantation delineates mouse embryogenesis. *bioRxiv* .

Cicek, O., Abdulkadir, A., Lienkamp, S. S., Brox, T. and Ronneberger, O. (2016). 3D U-Net: Learning Dense Volumetric Segmentation from Sparse Annotation. In *Medical Image Computing and Computer-Assisted Intervention – MICCAI 2016* (eds. S. Ourselin, L. Joskowicz, M. R. Sabuncu, G. Unal and W. Wells), volume 9901, pp. 424–432. Cham: Springer International Publishing.

Ciemerych, M. A. and Sicinski, P. (2005). Cell cycle in mouse development. *Oncogene* **24**, 2877–2898.

Fabrèges, D., Murtra, B. C., Moghe, P., Kickuth, A., Ichikawa, T., Iwatani, C., Tsukiyama, T., Daniel, N., Gering, J., Stokkermans, A. et al. (2023). Temporal variability and cell mechanics control robustness in mammalian embryogenesis. *bioRxiv* .

Hatamizadeh, A., Tang, Y., Nath, V., Yang, D., Myronenko, A., Landman, B., Roth, H. R. and Xu, D. (2022). UNETR: Transformers for 3D Medical Image Segmentation. In *2022 IEEE/CVF Winter Conference on Applications of Computer Vision (WACV)*, pp. 1748–1758. Waikoloa, HI, USA: IEEE.

Lalit, M., Tomancak, P. and Jug, F. (2022). Embedseg: Embedding-based instance segmentation for biomedical microscopy data. *Medical Image Analysis* **81**, 102523.

Lin, T.-Y., Maire, M., Belongie, S., Hays, J., Perona, P., Ramanan, D., Dollár, P. and Zitnick, C. L. (2014). Microsoft COCO: Common Objects in Context. In *Computer Vision – ECCV 2014* (eds. D. Fleet, T. Pajdla, B. Schiele and T. Tuytelaars), volume 8693, pp. 740–755. Cham: Springer International Publishing.

Lin, Z., Wei, D., Petkova, M. D., Wu, Y., Ahmed, Z., K, K. S., Zou, S., Wendt, N., Boulanger-Weill, J., Wang, X. et al. (2021). NucMM Dataset: 3D Neuronal Nuclei Instance Segmentation at Sub-Cubic Millimeter Scale. In *Medical Image Computing and Computer Assisted Intervention – MICCAI 2021* (eds. M. de Bruijne, P. C. Cattin, S. Cotin, N. Padoy, S. Speidel, Y. Zheng and C. Essert), volume 12901, pp. 164–174. Cham: Springer International Publishing.

Ortiz, R., de Medeiros, G., Peters, A. H. F. M., Liberali, P. and Rempfler, M. (2020). RDCNet: Instance Segmentation with a Minimalist Recurrent Residual Network. In *Machine Learning in Medical Imaging* (eds. M. Liu, P. Yan, C. Lian and X. Cao), volume 12436, pp. 434–443. Cham: Springer International Publishing.

Stringer, C., Wang, T., Michaelos, M. and Pachitariu, M. (2021). Cellpose: a generalist algorithm for cellular segmentation. *Nature Methods* **18**, 100–106.

Development • Supplementary information

Sugawara, K., Cevrim, C. and Averof, M. (2022). Tracking cell lineages in 3D by incremental deep learning. *eLife* **11**, e69380.

Tinevez, J.-Y., Perry, N., Schindelin, J., Hoopes, G. M., Reynolds, G. D., Laplantine, E., Bednarek, S. Y., Shorte, S. L. and Eliceiri, K. W. (2017). TrackMate: An open and extensible platform for single-particle tracking. *Methods* **115**, 80–90.

Tokuoka, Y., Yamada, T. G., Mashiko, D., Ikeda, Z., Hiroi, N. F., Kobayashi, T. J., Yamagata, K. and Funahashi, A. (2020). 3D convolutional neural networks-based segmentation to acquire quantitative criteria of the nucleus during mouse embryogenesis. *npj Systems Biology and Applications* **6**, 32.

Ulman, V., Maska, M., Magnusson, K. E. G., Ronneberger, O., Haubold, C., Harder, N., Matula, P., Matula, P., Svoboda, D., Radojevic, M. et al. (2017). An objective comparison of cell-tracking algorithms. *Nature Methods* **14**, 1141–1152.

Vijayan, A., Mody, T. A., Yu, Q., Wolny, A., Cerrone, L., Strauss, S., Tsiantis, M., Smith, R. S., Hamprecht, F. A., Kreshuk, A. et al. (2024). A deep learning-based toolkit for 3D nuclei segmentation and quantitative analysis in cellular and tissue context. *Development* **151**, dev202800.

Weigert, M., Schmidt, U., Haase, R., Sugawara, K. and Myers, G. (2020). Star-convex polyhedra for 3d object detection and segmentation in microscopy. In *The IEEE Winter Conference on Applications of Computer Vision (WACV)*.

Yang, L., Ghosh, R. P., Franklin, J. M., Chen, S., You, C., Narayan, R. R., Melcher, M. L. and Liphardt, J. T. (2020). NuSeT: A deep learning tool for reliably separating and analyzing crowded cells. *PLOS Computational Biology* **16**, e1008193.

Development • Supplementary information

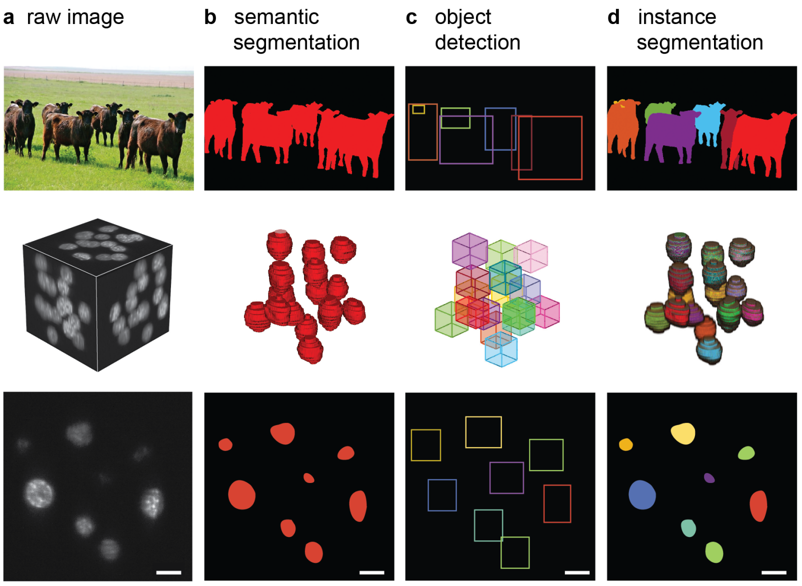

Fig. S1. Segmentation tasks applied to images of a pastoral scene (Lin et al., 2014) and of a mouse embryo. (A) Raw image to be segmented. From top to bottom, an image of cows in a pasture, maximum intensity projections of 3D image of 16-cell mouse embryo, a z-slice from the 3D image. 3D image has dimensions (83.4 μm, 83.4 μm, 68 μm). Scale bar: 10 μm. (B) Semantic segmentation for images in (A). (C) Object detection for images in (A). (D) Instance segmentation for images in (A).

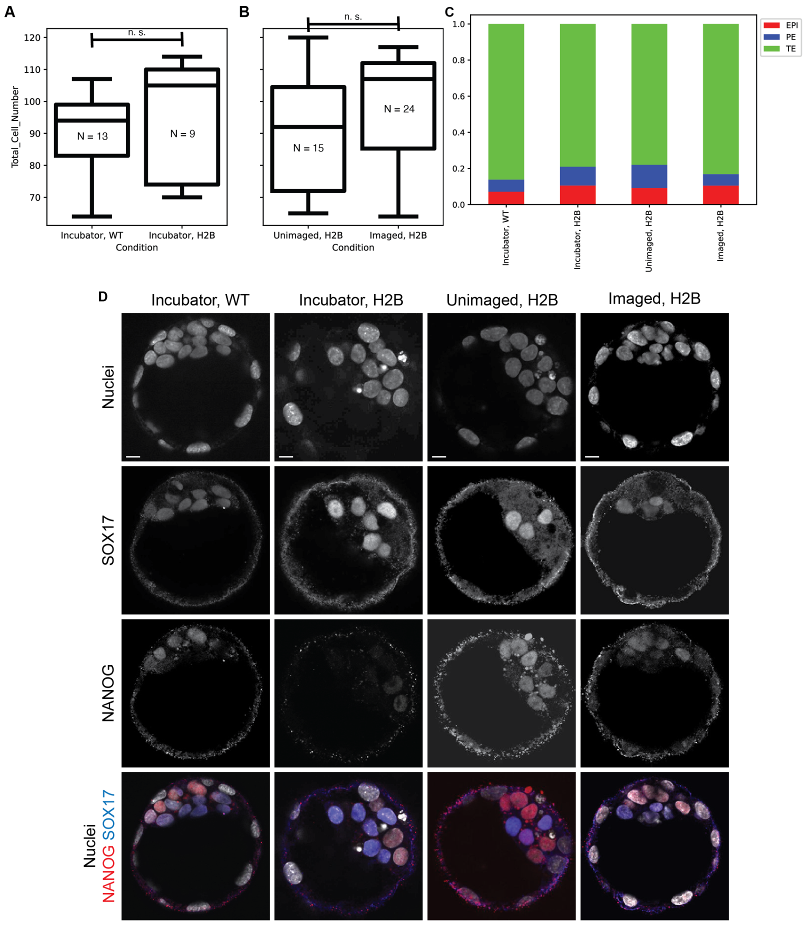

Fig. S2. **Comparison of lineage composition and total cell number for differ-ent experimental conditions and mouse lines.** (A,B) Statistical comparison of total cell number for Incubator wild-type versus Incubator H2B ($p = 0.50$) and for Unimaged H2B versus Imaged H2B ($p = 0.08$). All embryos were cultured from E2.5 to E4.5. N = 13 (Incubator, WT), 9 (Incubator, H2B), 15 (Unimaged, H2B), 24 (Imaged, H2B). (C) Average proportion of EPI, PE and TE fates for all conditions: Imaged H2B, Unimaged H2B, Incubator, WT and Incubator, H2B. Fate proportions are comparable for each of the two comparisons. All embryos were cultured from E2.5 to E4.5. Sample sizes (N) same as listed in (A,B). (D) Representative slices of 3D images of immunostained embryos for each of the conditions in (C). Scale bar: 10 μm.

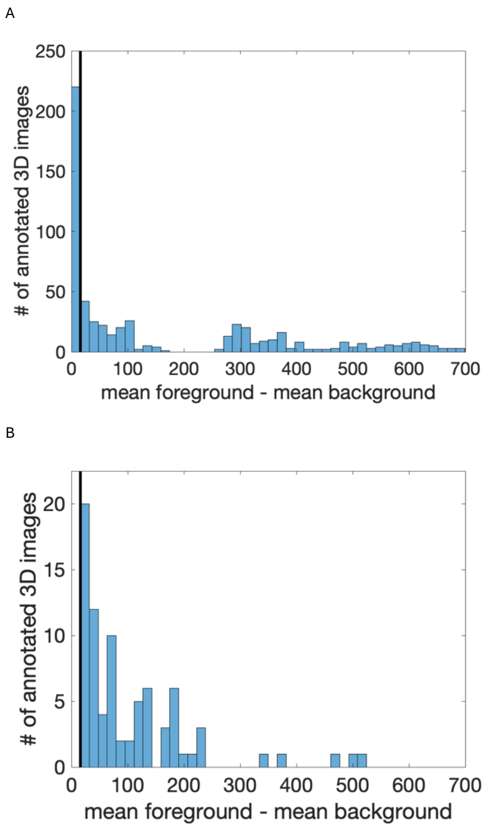

Fig. S3. **Foreground-background intensity difference of each image in the Blas-toSPIM dataset.** (A-B) Histogram, for annotated images in the original BlastoSPIM set and in the corrected late blastocyst segmentations, respectively, of the difference between mean foreground intensity and the mean background intensity. Black line: Cutoff for separat-ing low foreground-background intensity difference from moderate foreground-background intensity difference in the original BlastoSPIM dataset.

Development • Supplementary information

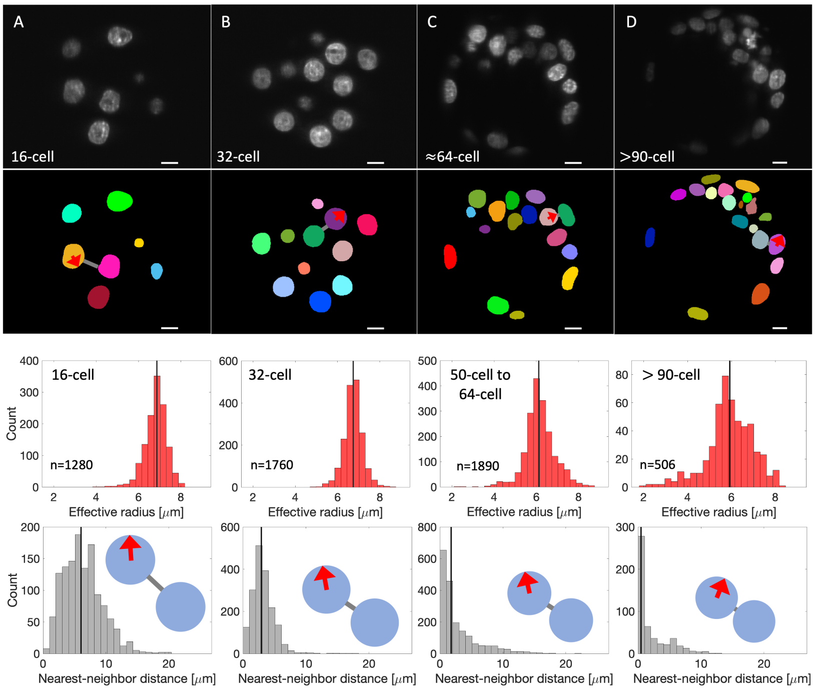

Fig. S4. Nearest-neighbor distances between nuclei decrease dramatically during development. (A-D) Example z-slices and quantification for 16-cell (A), 32-cell (B), 50-to- 64-cell (C), and >90-cell (D) embryos. The first two rows contain images and corresponding annotations. Each red arrow indicates the nucleus's effective radius, the radius of a sphere of equivalent volume. The gray lines indicate examples of shortest surface-to-surface distance. The third and fourth rows show that the effective radius and the shortest surface-to-surface distance decrease during development. Illustrations in the bottom histograms show that the latter decreases more than the former. Median of histogram in black. Scale bar: 10 μm.

Development • Supplementary information

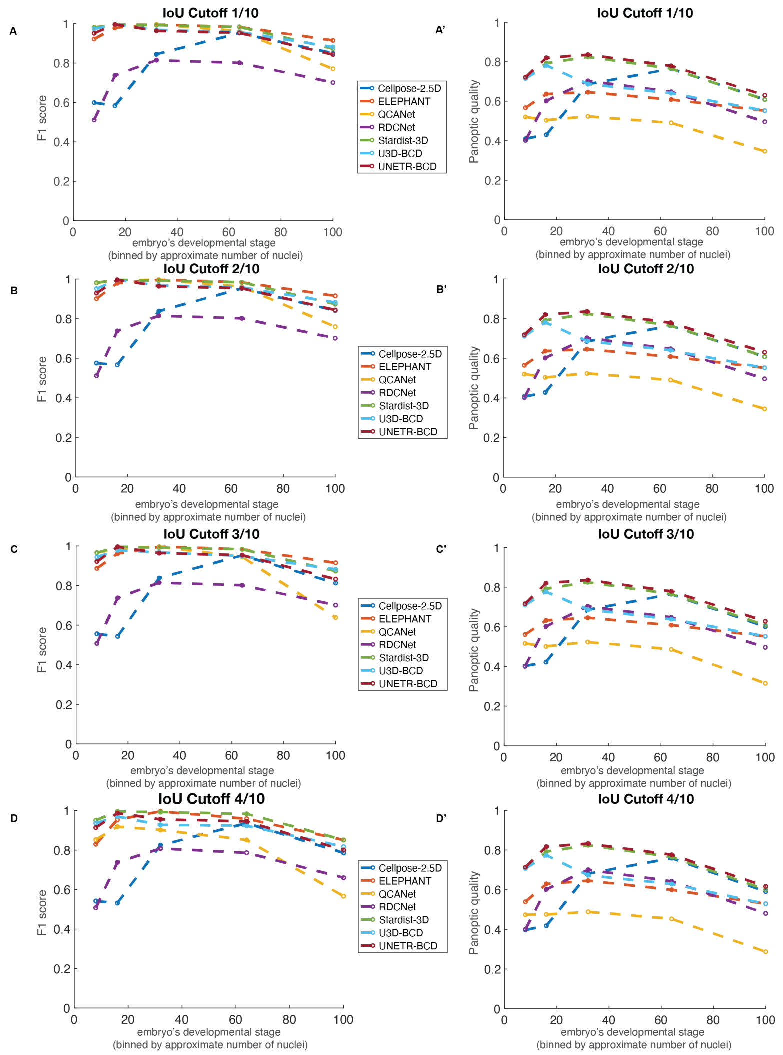

Fig. S5. F_1 **score and panoptic quality across IoU thresholds (0.1-0.4) for seven methods. Analogous to Fig. 2(A,B).**

(A,A') $F1$ score and panoptic quality, respec-tively, for an IoU cutoff of 0.1. (B,B') Same as in (A,A'), for an IoU cutoff of 0.2. (C,C') Same as in (A,A'), for an IoU cutoff of 0.3. (D,D') Same as in (A,A'), for an IoU cutoff of 0.4. Note that Figure 2(A,B) are based on an IoU cutoff of 0.5.

Development • Supplementary information

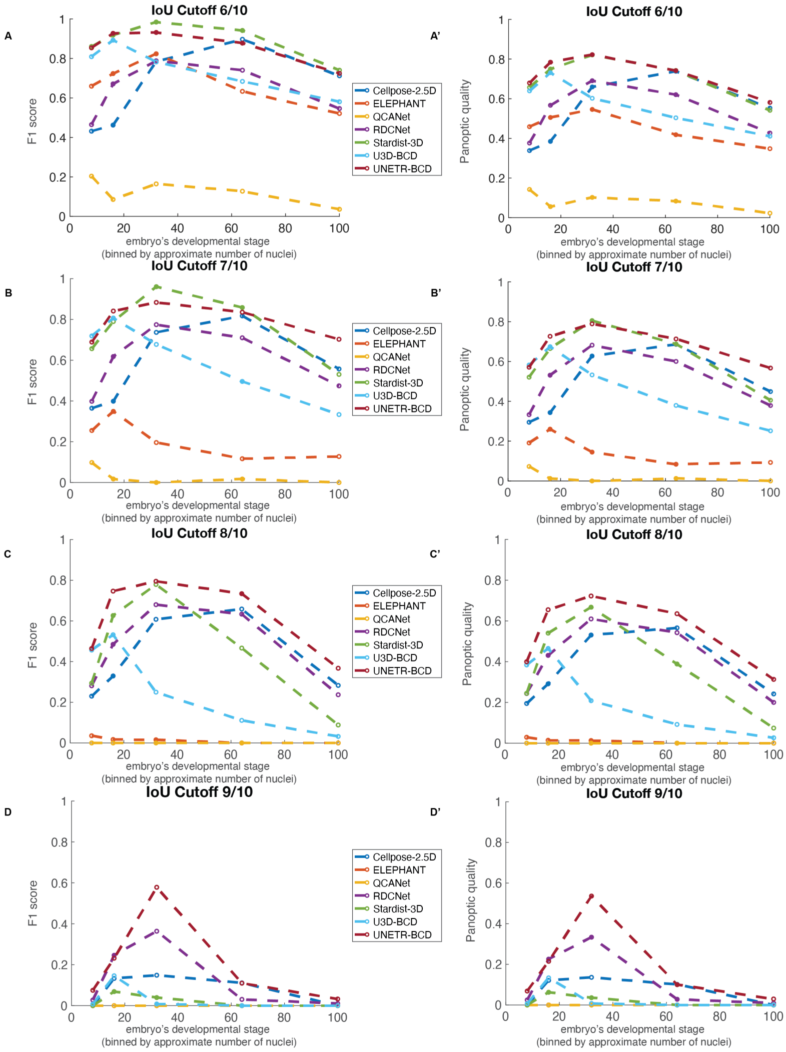

Fig. S6. F_1 **score and panoptic quality across IoU thresholds (0.6-0.9) for seven methods. Analogous to Fig. 2(A,B).** (A,A') F1 score and panoptic quality, respec-tively, for an IoU cutoff of 0.6. (B,B') Same as in (A,A'), for an IoU cutoff of 0.7. (C,C') Same as in (A,A'), for an IoU cutoff of 0.8. (D,D') Same as in (A,A'), for an IoU cutoff of 0.9. Note that Figure 2(A,B) are based on an IoU cutoff of 0.5.

Development • Supplementary information

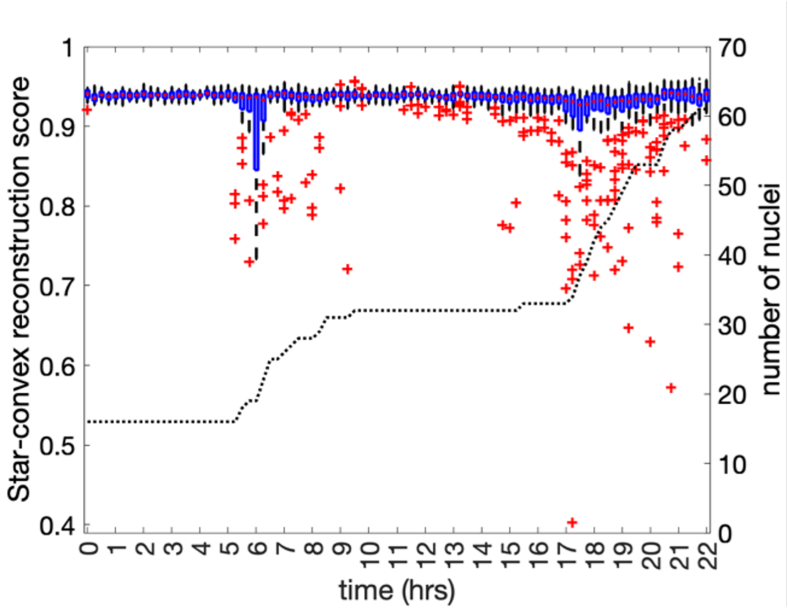

Fig. S7. **Quantifying how well star-convex approximation applies to nuclear shapes in ground-truth time series data.** We fit each nucleus to a star-convex shape, using 128 rays. For a single embryo, for which we have annotated ground truth for 89 con-secutive timepoints (time points acquired every 15 minutes), we plot a box for each time to illustrate how well this fit performs, in terms of IoU. When all nuclei are in interphase, the star-convex fit performs quite well, at more than 90 percent IoU between the ground truth and the model-generated instance. During the transition from the 16-cell stage to the 32-cell stage and from the 32-cell stage to the 64-cell stage, the fit quality degrades. A small number of nuclei, about five in this time series cannot be fit by a star-convex shape, result-ing in an IoU of less than 40 percent. We expect that the outlier nuclei (red) – which are not well fit by a star-convex shape – are likely mitotic, most likely in either metaphase or anaphase when the shape of the condensed chromatin is often complex. Black dashed line: the number of nuclei versus time.

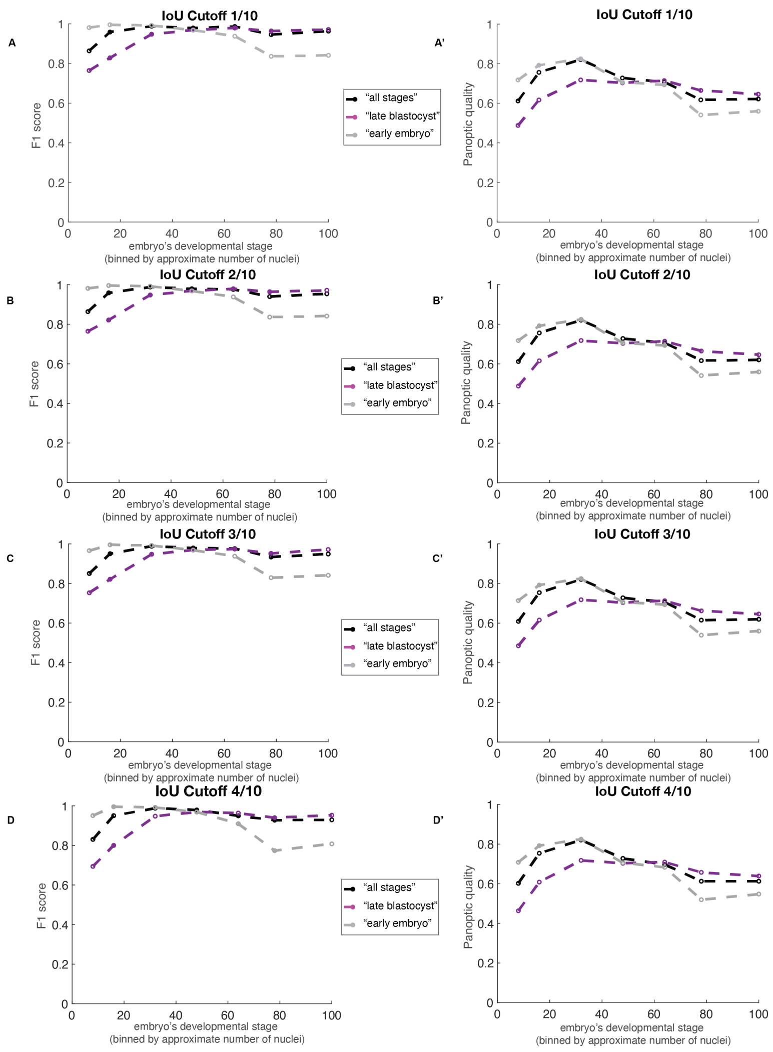

Fig. S8. **F_1 score and panoptic quality across IoU thresholds (0.1-0.4) for early embryo, late blastocyst, and all stages models. Analogous to Fig. 3(A,B).**(A,A') F_1 score and panoptic quality, respectively, for an IoU cutoff of 0.1. (B,B') Same as in (A,A'), for an IoU cutoff of 0.2.(C,C') Same as in (A,A'), for an IoU cutoff of 0.3. (D,D') Same as in (A,A'), for an IoU cutoff of 0.4. Note that Figure 3(A,B) are based on an IoU cutoff of 0.5.

Development • Supplementary information

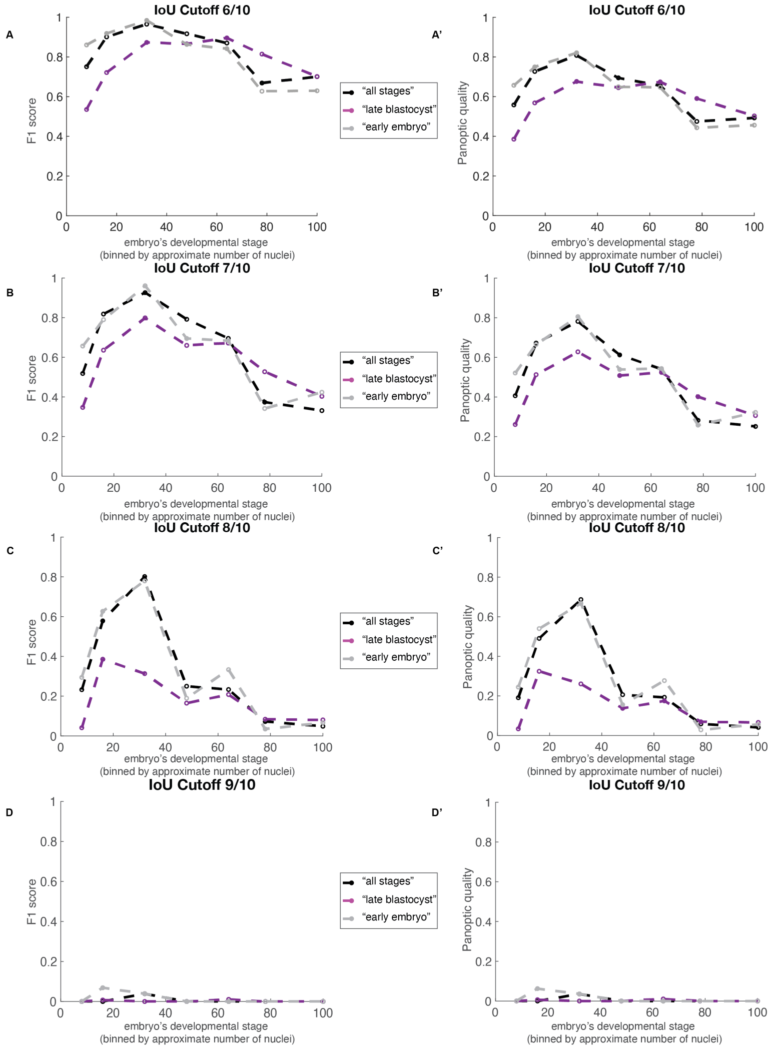

Fig. S9. **F_1 score and panoptic quality across IoU thresholds (0.6-0.9) for early embryo, late blastocyst, and all stages models. Analogous to Fig. 3(A,B).**(A,A') F_1 score and panoptic quality, respectively, for an IoU cutoff of 0.6. (B,B') Same as in (A,A'), for an IoU cutoff of 0.7. (C,C') Same as in (A,A'), for an IoU cutoff of 0.8. (D,D') Same as in (A,A'), for an IoU cutoff of 0.9. Note that Figure 3(A,B) are based on an IoU cutoff of 0.5.

Development • Supplementary information

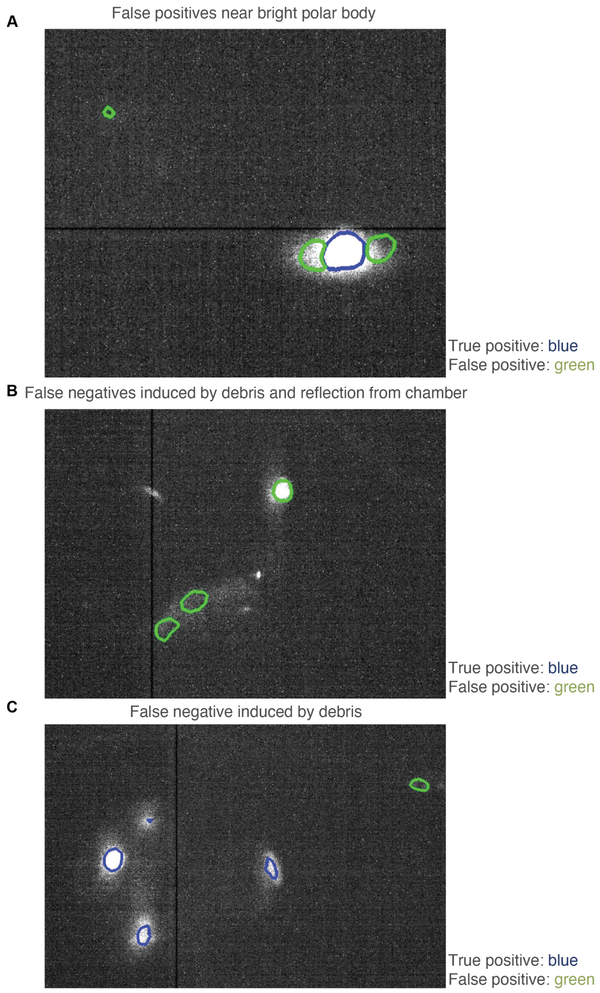

Fig. S10. Qualitative evaluation of failure modes for late blastocyst model on images of early embryos, particularly those with low foreground-background intensity difference. Blue: Model prediction which is a true positive. Green: Model pre-diction which is a false positive. (A) z-slice containing only a polar body. Note that the late blastocyst model predicts a couple of false positives around the polar body. (B) z-slice in which debris and a reflection from the image chamber are falsely segmented into instances.(C) z-slice in which a false positive is detected away from the embryo (see region with true positives).

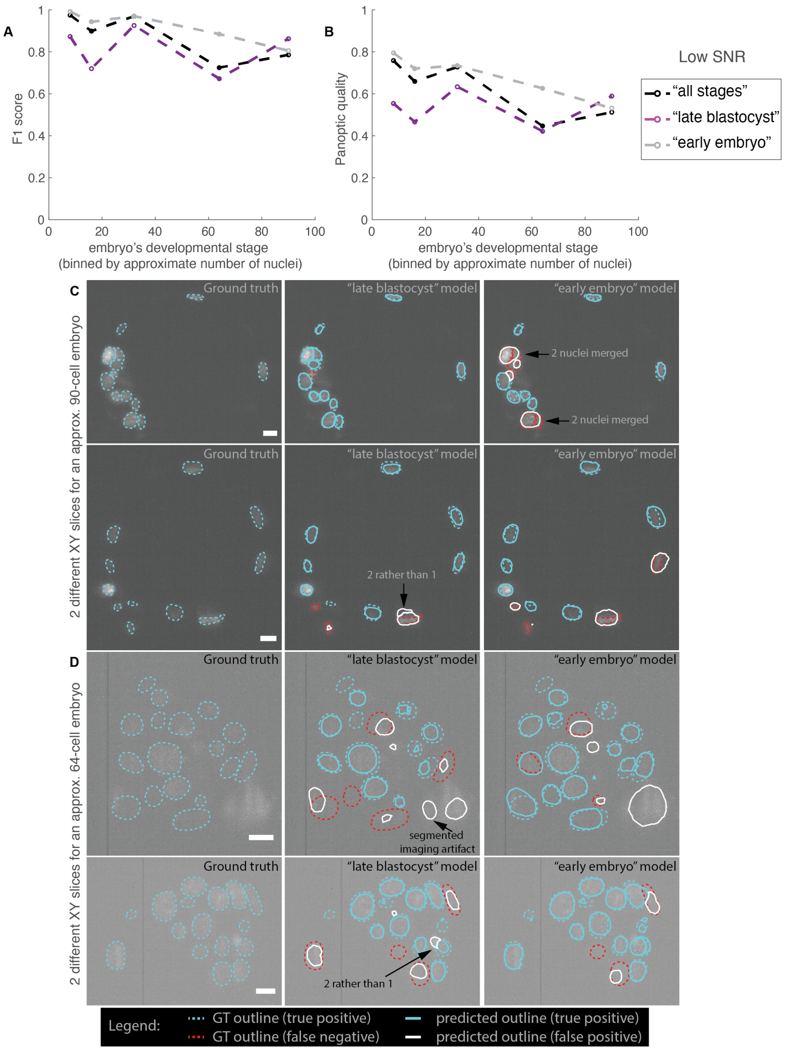

Fig. S11. Quantitative and qualitative evaluation of failure modes for the early embryo model and the late blastocyst model on images with particularly low foreground-background intensity difference. (A,B) F_1-score and panoptic quality, respectively, for the low foreground-background intensity difference test set, binned by developmental stage.(C,D) Representative xy slices from an ≈ 90-cell embryo and an ≈ 64-cell embryo, respectively. Results from the early embryo model in (C) illustrate its tendency to improperly merge closely juxtaposed nuclei into a single instance. Results from the late blas-tocyst model in (C,D) illustrate its tendency to produce false positives, sometimes in regions with nuclei which are blurred or haloed due to imaging artifacts. It is worth noting that these images, particularly (D), have extraordinarily low foreground-background intensity difference, which makes even manual annotation difficult. The results in this supplemental figure, thus, represent some of the most difficult test images to segment.

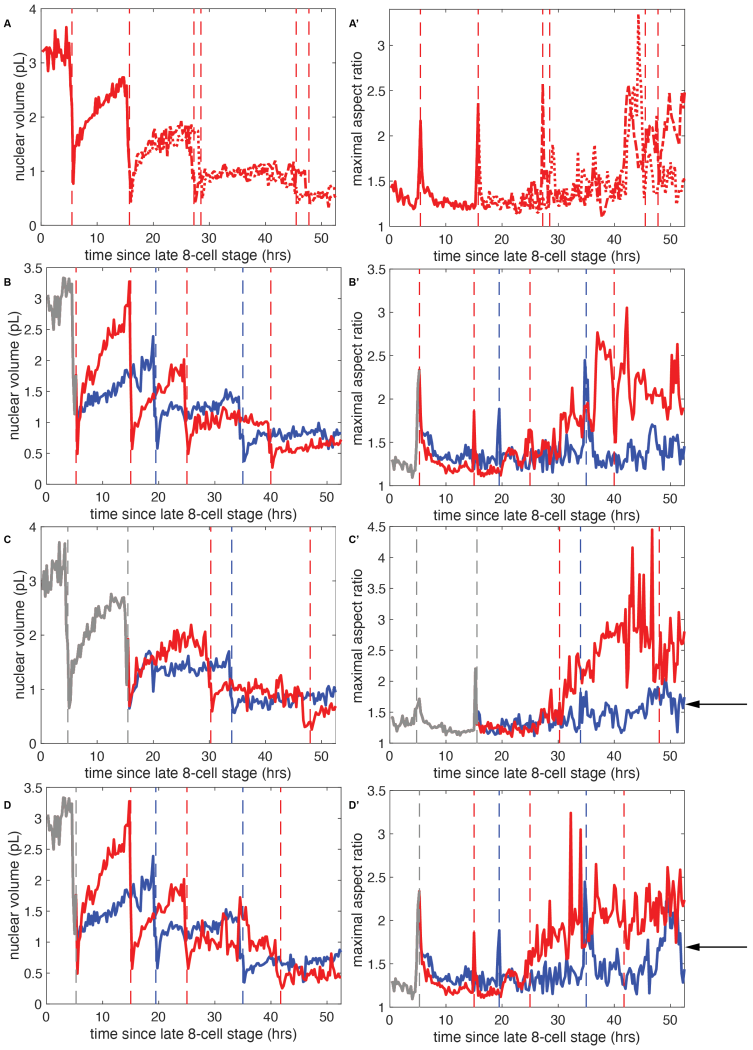

Fig. S12. Dynamics of nuclear aspect ratios and nuclear volumes from Fig. 5(A). (A,A') Nuclear volume and nuclear aspect ratio, respectively, for two paths in the lineage tree from the same root. When the paths split at a division, one resulting daughter's line is dashed, while the other's remains solid. Vertical dashed lines indicate division events.(B,B') Nuclear volume and nuclear aspect ratio, respectively, for two paths illustrated in Fig. 5(B). Colors as in the tree in Fig. 5(A). (C,C') Nuclear volume and nuclear aspect ratio, respectively, for two paths from the same root to leaves with different fates (one ICM, one TE). Arrow indicates increased aspect ratio of ICM nucleus towards the end of the time lapse. (D,D') Same as in (C,C'), but for two different paths.

Development • Supplementary information

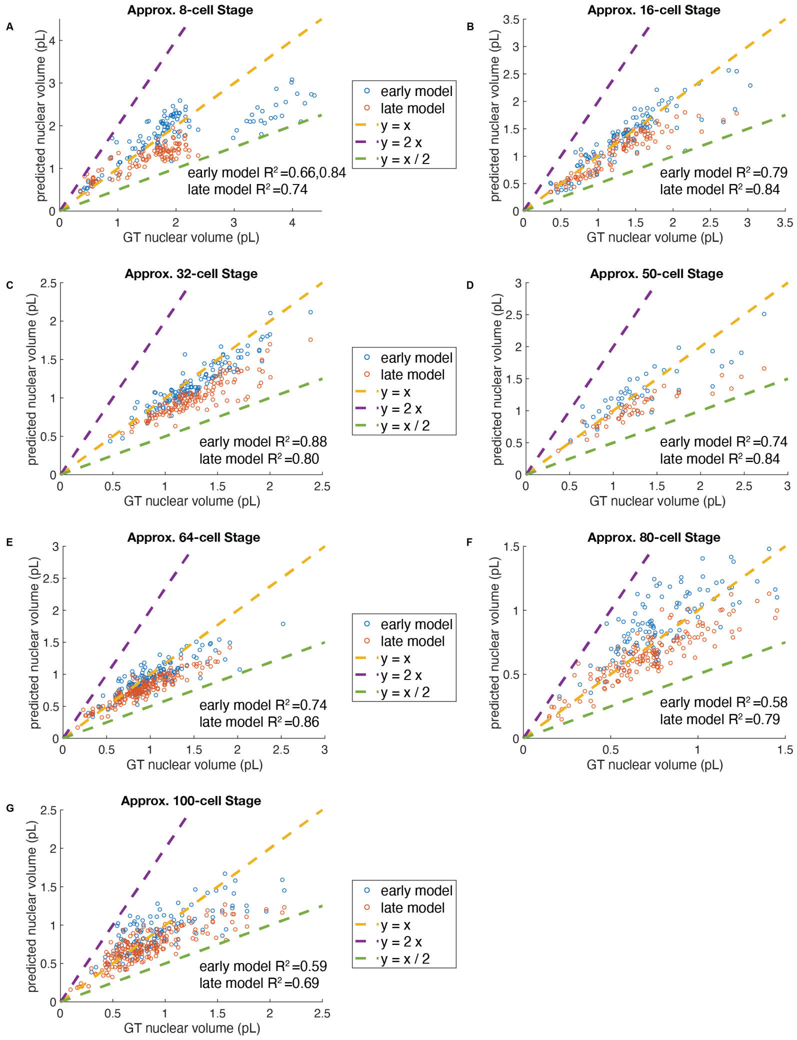

Fig. S13. Comparing ground-truth and model-predicted nuclear volumes across embryonic stages. For the combined ground-truth set used in Fig. 3(A,B), if a ground-truth instance is matched to a model-predicted instance by an IoU of at least 0.5, we plot the ground-truth nuclear volume against the predicted nuclear volume. Each panel (A-G) is labelled with the corresponding developmental stage. See legend for meaning of dashed lines. Note that the large ground-truth instances at the 8-cell stage correspond to a couple of ground-truth images in which an annotator overestimated nuclear sizes because of low foreground-background intensity difference. Encouragingly, our model predicted volumes for these instances which are closer to range of the rest of ground-truth volumes. For the 8-cell stage, the second R^2 value is for the set with the overestimated ground-truth volumes removed.

Development • Supplementary information

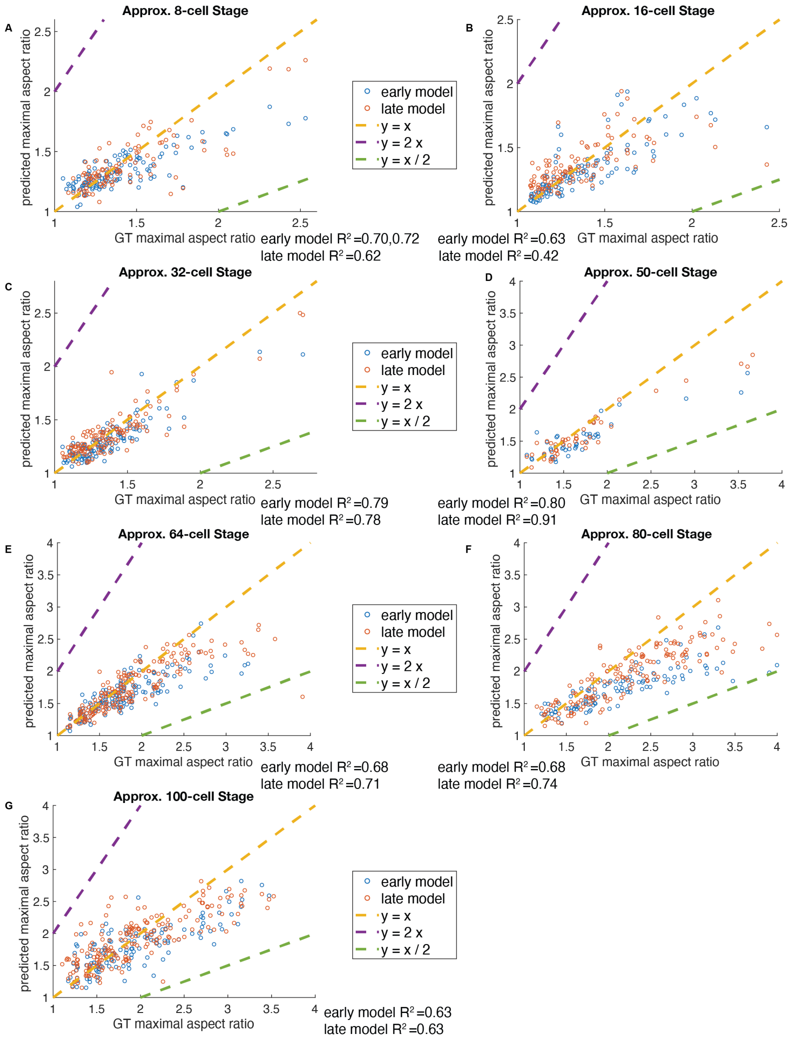

Fig. S14. Comparing ground-truth and model-predicted aspect ratios across embryonic stages. For the combined ground-truth set used in Figure 3(A,B), if a ground-truth instance is matched to a model-predicted instance by an IoU of at least 0.5, we plot the ground-truth nuclear aspect ratio against the predicted nuclear aspect ratio. Each panel (A-G) is labelled with the corresponding developmental stage. See legend for meaning of dashed lines.

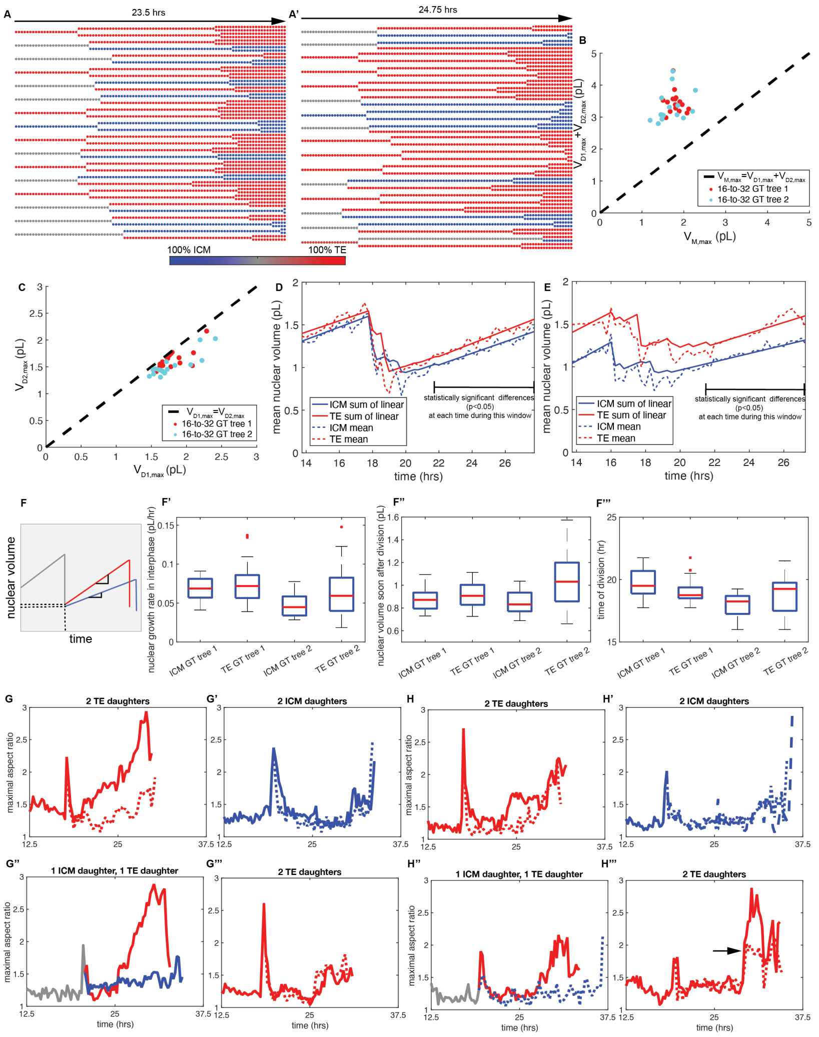

Fig. S15. Nuclear volumes and aspect ratios in 2 ground-truth lineages. (A,A') Two lineages with ground-truth nuclear annotation and tracking. (B) Plot analogous to Figure 5(D), for the lineages in (A,A') (one in cyan, one in red). Each point represents one mother nucleus from the 16-cell stage giving rise to two daughter nuclei at the 32-cell stage.(C) Plot analogous to Fig. 5(E), for the two ground-truth lineages (one in cyan, one in red). (D) Comparison of TE and ICM nuclear volumes during the 32-cell stage (based on rank sum test at each time). Dashed lines: mean nuclear volumes for the ICM and TE in (A). Solid lines: result of fitting lines to inter-division nuclear trajectories, then averaging for the ICM and TE separately. (E) Same as in (D) but for the lineage in (A'). (F) By fitting a line to each inter-division interval at the 32-cell stage, we extract the value of the linear fit immediately after the division (horizontal dashed lines), the growth rate (indicated by solid black lines), and the time of division (vertical dashed line). (F', F'', F''') Comparisons between ICM and TE linear fits. (G-G''') Example nuclear aspect ratios for the lineage in (A) Each segment is colored as in (A). If two daughters are of same fate, one is plotted as a dashed line. Lines end when next division occurs. (H-H''') Same as (G-G''') but for the lineage in (A') Black arrow: change in nuclear aspect ratio.

Development • Supplementary information

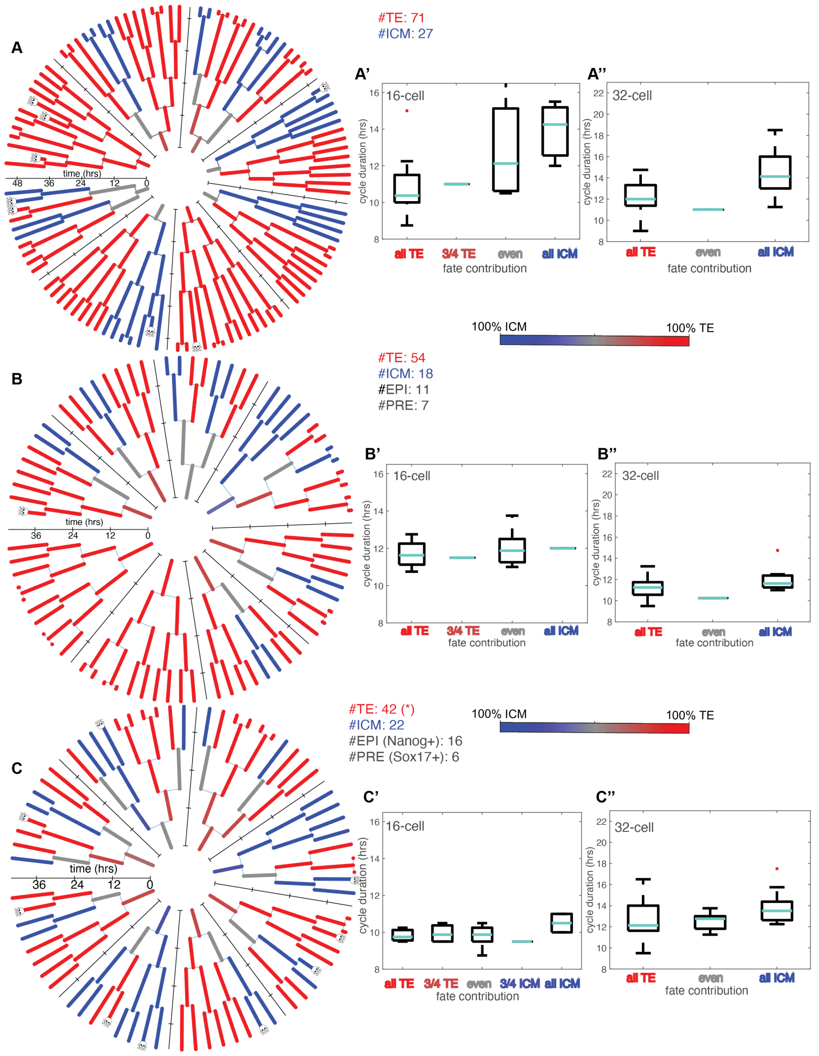

Fig. S16. Relationship between ICM/TE fate contribution and cell cycle dura-tions for H2B-miRFP720 embryos. (A) Lineage tree of an H2B-miRFP720-expressing embryo. Color indicates eventual contributions to the ICM and TE. Skull: cell death event.(A') Box plot of cell cycle durations, grouped by eventual fate contributions, at the 16-cell stage. (A") Box plot of cell cycle durations, grouped by eventual fate contributions, at the 32-cell stage. (B-B") Same as in (A-A"), but for a different H2B-miRFP720-expressing embryo. (C-C") Same as in (A-A"), but for a different H2B-miRFP720-expressing embryo.(B,C) include counts of cell fates from fixing and staining the embryo after the final time-point in live imaging. Asterisk in (C) refers to a slight discrepancy in TE count between the endpoint of live imaging and the fixed embryo – likely explained by cell extrusion happening at the end of live imaging.

Development • Supplementary information

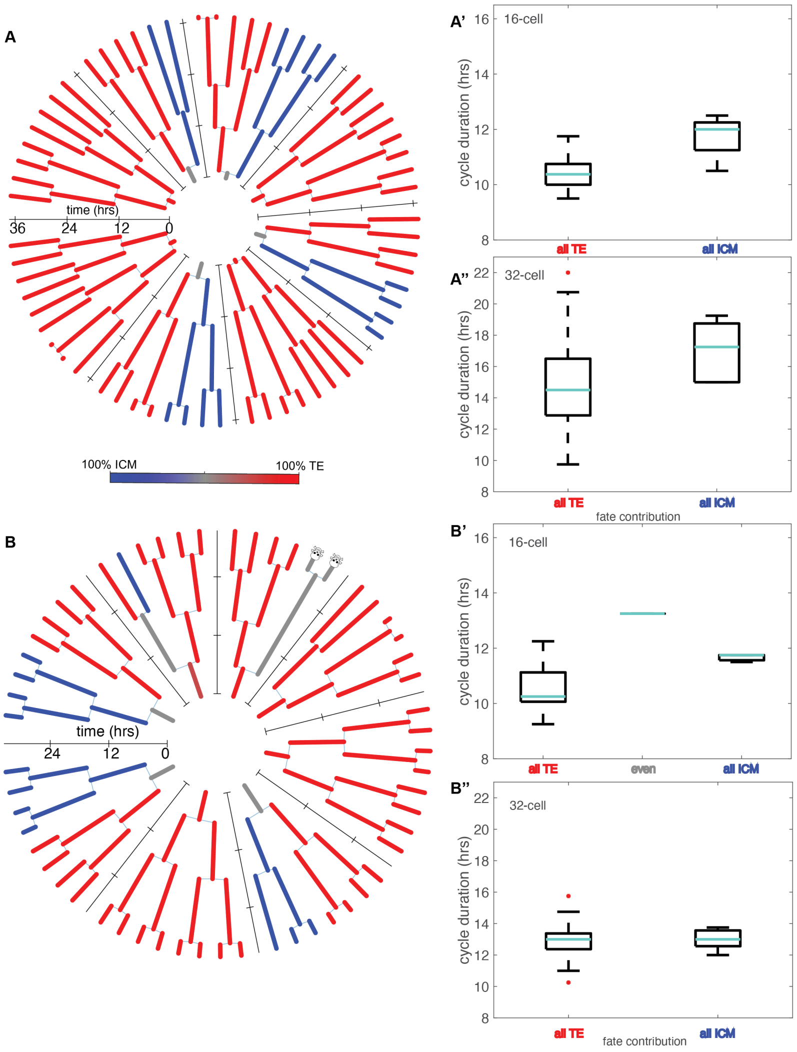

Fig. S17. Relationship between ICM/TE fate contribution and cell cycle durations for H2B-miRFP720;Cdx2-eGFP embryos. (A) Lineage tree of an H2B-miRFP720;Cdx2-eGFP embryo. Color indicates eventual contributions to the ICM and TE. Skull: cell death event. Cell fate assigned based on method outlined in Fig. 5(G). (A') Box plot of cell cycle durations, based on fate contributions, at the 16-cell stage. (A") Box plot of cell cycle durations, based on fate contributions, at the 32-cell stage. (B-B") Same as in (A-A"), but for a different H2B-miRFP720;Cdx2-eGFP embryo.

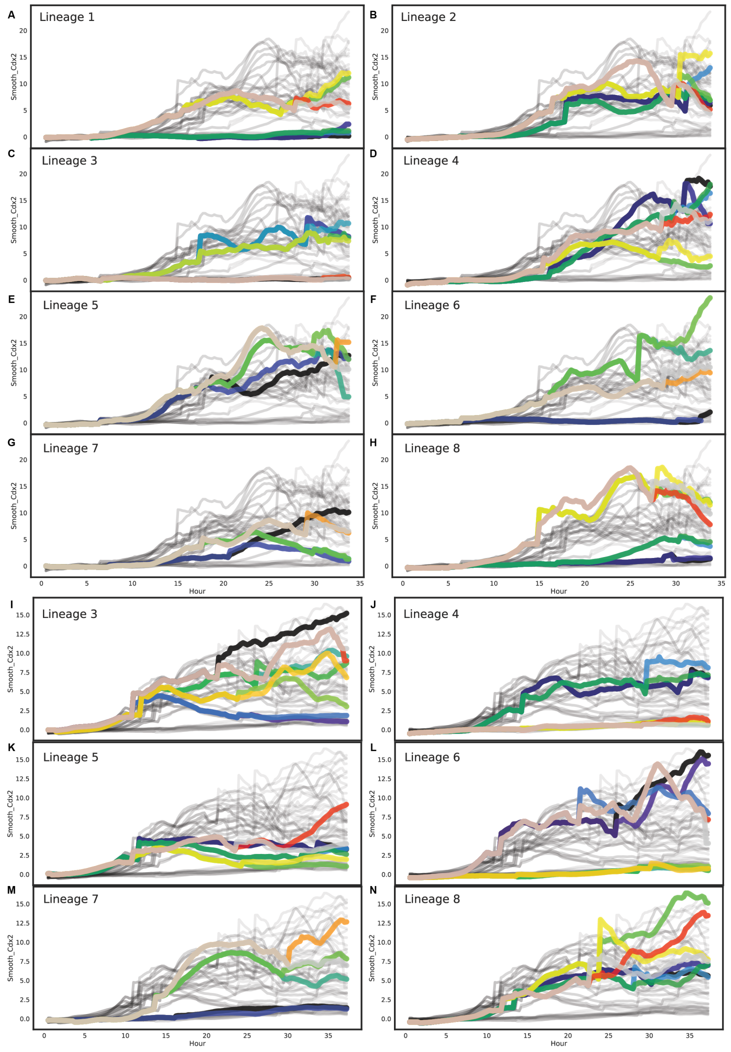

Fig. S18. Cdx2-eGFP traces from nuclear tracks from two different embryos expressing both H2B-miRFP720 and Cdx2-eGFP. (A-H) Each plot represents the Cdx2-eGFP trace from a nucleus at the 8-cell stage and all of its progeny. Traces from other nuclei in the same embryo in gray. This embryo is the same as in Fig. S17(B). (I-N) Each plot represents the Cdx2-eGFP trace from a nucleus at the 8-cell stage and all of its progeny. The other two lineage trees from this same embryo are in Fig. 5(F,F'). This embryo is the same as in Fig. S17(A).

Table S1. Tools for 3D nuclear segmentation. Underlined: methods benchmarked here

Tool name	Base network	Network output	Loss metrics	Post-processing
Cellpose (Stringer et al., 2021)	2D U-Net with residual blocks, style transfer	horizontal/ vertical gradient maps, cell probability map	L2 loss (gradients), cross-entropy loss (cell probability)	probability threshold and test-time enhancements
QCANet (Tokuoka et al., 2020)	Two 3D U-Nets, hyper-parameter tuning by Bayesian optimization	Semantic segmentation, nucleus center detection	Dice loss	Reinterpolation and marker-based watershed
NuSeT (Yang et al., 2020)	2D U-Net integrated with Region Proposal Network (RPN)	Semantic segmentation, bounding box with score	cross-entropy loss + Dice loss (segmentation), class loss and regression loss (detection)	watershed and 3D reconstitution from 2D slices
Stardist (Weigert et al., 2020)	3D ResNet or 3D U-Net	radial distances to object boundary, object probability (OP) with distance transform	cross-entropy loss (OP), OP-weighted mean absolute error with regularization (radial distances)	OP threshold and non-maximum suppression
RDCNet (Ortiz et al., 2020)	3D recurrent block with stacked dilated convolutions	semantic classes, semi-convolutional embeddings	Embedding soft jaccard (ESJ) loss	Margin thresholds, Hough voting
EmbedSeg (Lalit et al., 2022)	3D Branched ERF-Net	pixel embeddings, clustering bandwidth, seed probability	Lovász-Softmax loss + seed loss + smoothness loss	seed probability threshold, cluster bandwidth threshold
U3D-BCD (Lin et al., 2021)	3D U-Net with residual blocks substituted for convolutional layers	foregrounds masks, instance contours, signed-distance-transform map	Weighted sum of cross-entropy loss and dice loss for foreground and contour; mean-squared error for signed distance	seed detection via threshold on foreground probability and distance value, marker-controlled watershed
UNETR-BCD (Hatamizadeh et al., 2022)	Stack of transformers connected to 3D CNN-based decoder	foregrounds masks, instance contours, signed-distance-transform map	Weighted sum of cross-entropy loss and dice loss for foreground and contour; mean-squared error for signed distance	seed detection via threshold on foreground probability and distance value, marker-controlled watershed
ELEPHANT (Sugawara et al., 2022)	3D U-Net (Cicek et al., 2016)	probability maps for nucleus center, nucleus periphery, and background	sum of (i) class-weighted negative log-likelihood loss, (ii) one minus the dice coefficient for nucleus center voxels, (iii) term penalizing roughness in nucleus center areas	edge subtraction (background class) from nucleus center probablities, thresholding on nucleus center probability, enlargement of ellipses

Development • Supplementary information

Table S2. Ground-truth, three-dimensional annotations of nuclei in animals. Also, see the plant dataset in (Vijayan et al., 2024). *The entire ground-truth dataset used in (Tokuoka et al., 2020) contains more than 6000 time-series of early mouse embryos, of which only 165 have been made publicly available.

Name	Microscopy	Nuclear Labeling	Sample	Image Count	Network results
NucMM-Z (Lin et al., 2021)	Serial-section electron microscopy	N/A	Zebrafish brain	1	Cellpose3D, Stardist-3D, U3D-BCD
NucMM-M (Lin et al., 2021)	Micro-CT	N/A	Mouse visual cortex	1	Cellpose3D, Stardist-3D, U3D-BCD
BBBC050* (Tokuoka et al., 2020)	Confocal microscopy	H2B-mRFP1, H2B-mCherry	Pre-implantation mouse embryo from the pro-nuclear stage to 53-cell stage	165	QCANet, 3D U-Net, 3D Mask R-CNN
C. elegans developing embryo(Ulman et al., 2017)	Confocal microscopy	histone-GFP	*C. elegans* embryo between 2-cell stage and ≈300-cell stage	9	QCANet, 3D U-Net, 3D Mask R-CNN
Platynereis-Nuclei-CBG(Lalit et al., 2022)	Light-Sheet Microscopy	Fluorescent nuclear tracer injected	*Platynereis dumerilii* embryo between 0 and 16 hours post-fertilization	9	Cellpose3D, Stardist-3D, EmbedSeg
Platynereis-ISH-Nuclei-CBG (Lalit et al., 2022)	Confocal Microscopy	DAPI	*Platynereis dumerilii* specimins 16 hours post-fertilization	2	Cellpose3D, Stardist-3D, EmbedSeg
Parhyale hawaiensis-Nuclei (Weigert et al., 2020)	Confocal Microscopy	H2B-eGFP	*Parhyale hawaiensis* embryo between 46 hours post-amputation (hpa) and 110 hpa	6	U-Net, Stardist-3D, Cellpose3D, EmbedSeg
C. elegans-Nuclei (Weigert et al., 2020)	Confocal Microscopy	DAPI	*C. elegans* embryo at the 558-cell stage	28	U-Net, Stardist-3D
Mouse-Skull-Nuclei-CBG (Lalit et al., 2022)	Confocal Microscopy	DAPI	Nuclei from the skull of developing mouse embryos	2	Cellpose3D, Stardist-3D, EmbedSeg
Peri-implantation mouse embryos (Bondarenko et al., 2022)	Confocal Microscopy	Antibody staining	Peri-implantation mouse embryos	35	3D U-Net

Development • Supplementary information

Table S3. Segmentation of Polar Bodies. There were 13 images in which a total of 15 polar bodies were labelled as such. All the methods detected all the polar bodies (except one that Cellpose missed at IoU=0.1) but with varying degrees of success in how well they were segmented. At IoU=0.5, most methods missed a few, except for RDCNet which was able to detect them all. We hypothesize that RDCNet which uses much fewer parameters given its recursive design, is better able to learn to detect polar bodies using the few examples in the training set.

**Method name**	**Average IoU**	**Misses (IoU<0.1)**	**Misses (IoU<0.5)**	**FP (IoU=0.5)**
Cellpose	0.47	1	7	6
QCANet	0.35	0	14	14
Stardist	0.66	0	2	2
RDCNet	0.79	0	0	0
U3D-BCD	0.66	0	4	4
UNETR-BCD	0.70	0	2	2
ELEPHANT	0.60	0	5	5

Table S4. Segmentation of H2B signal in mitotic cells. For mitotic nuclei, there were only 3 late-stage images (2 at the 64-cell stage, and 1 at 100-cell stage) with 6 examples. Nuclei were considered mitotic if they were in metaphase or anaphase. For these late-stage embryos, there are many small nuclei tightly packed together. All the methods missed at least one mitotic nuclei at IoU=0.5 with UNETR-BCD performing the best with only one.

**Method name**	**Average IoU**	**Misses (IoU<0.1)**	**Misses (IoU<0.5)**	**FP (IoU=0.5)**
Cellpose	0.52	1	2	2
QCANet	0.27	1	5	5
Stardist	0.46	1	2	2
RDCNet	0.37	0	4	4
U3D-BCD	0.41	1	4	4
UNETR-BCD	0.65	0	1	1
ELEPHANT	0.37	0	4	4

Table S5. Evaluation of Trackmate (Tinevez et al., 2017) and our method on the time-lapse images of the embryo in Figure 5(A). We evaluated the lineage trees produced by each technique against the hand-corrected lin-eage. A true positive indicates a correctly matched pair of nuclei between two frames, a false positive indicates an incorrectly matched pair, and a false neg-ative indicates a missing pair. We include the results for Trackmate both with and without pre-registration of label images. Our tracking method without hand-corrections outperformed Trackmate with its highest-performing param-eter combination and pre-registered inputs. See Methods for a discussion of Trackmate parameter optimization.

**Method name**	**TP**	**FP**	**FN**	**F1-score**
Ours	9379	39	38	0.9959
Trackmate unregistered	4516	4664	276	0.6464
Trackmate registered	8929	432	95	0.9713

Development • Supplementary information
